# Improving the acoustic performance of flexible polyurethane foam using biochar modified by (3-aminopropyl)trimethoxysilane coupling agent

**DOI:** 10.1038/s41598-024-68039-w

**Published:** 2024-08-08

**Authors:** Ahmed Abdelhamid Maamoun, Ramadan M. Abouomar, Tarek M. El-Basheer, Mostafa A. Azab, ElSayed G. Zaki, Shymaa M. Elsaeed, Ahmed Elkhateeb

**Affiliations:** 1 Department of Engineering Physics and Mathematics, Chemistry Division, Faculty of Engineering, Ain Shams University, 1 EL-Sarayat Street—Abdo Basha Sq., Cairo, 11517 Egypt; 2 Egyptian Petroleum Research Institute, Nasr City, Cairo, 11727 Egypt; 3 Department of Acoustics, Mass and Force Metrology Division, National Institute of Standards (NIS), El-Sadat Street, El-Haram, El-Giza, 12211 Egypt; 4 Department of Architecture, Faculty of Engineering, Ain Shams University, 1 EL-Sarayat Street—Abdo Basha Sq., Cairo, 11517 Egypt

**Keywords:** Flexible polyurethane, Eggshell waste, Biochar, Composites, Sound absorption, Biomaterials, Materials science, Environmental chemistry, Polymer chemistry

## Abstract

This study aims to investigate the potential of integrating natural biochar (BC) derived from eggshell waste into flexible polyurethane (FPU) foam to enhance its mechanical and acoustic performance. The study explores the impact of incorporating BC at various weight ratios (0.1, 0.3, 0.5, and 0.7 wt. %) on the properties of the FPU foam. Additionally, the effects of modifying the BC with (3-aminopropyl)trimethoxysilane (APTMS) at different ratios (10, 20, and 30 wt. %) and the influence of diverse particle sizes of BC on the thermal, mechanical, and acoustic characteristics of the FPU composite are investigated. The functional groups, morphology, and elemental composition of the developed FPU composites are analyzed using Fourier-transform infrared spectroscopy (FTIR), field-emission scanning electron microscopy (FESEM), and energy-dispersive X-ray (EDX) techniques. Characteristics such as density, gel fraction, and porosity were also assessed. The results reveal that the density of FPU foam increased by 4.32% and 7.83% while the porosity decreased to 50.22% and 47.05% with the addition of 0.1 wt. % of unmodified BC and modified BC with 20 wt. % APTMS, respectively, compared to unfilled FPU. Additionally, the gel fraction of the FPU matrix increases by 1.91% and 3.55% with the inclusion of 0.1 wt. % unmodified BC and modified BC with 20 wt. % APTMS, respectively. Furthermore, TGA analysis revealed that all FPU composites demonstrate improved thermal stability compared to unfilled FPU, reaching a peak value of 312.17°C for the FPU sample incorporating BC modified with 20 wt. % APTMS. Compression strength increased with 0.1 wt. % untreated BC but decreased at higher concentrations. Modifying BC with 20% APTMS resulted in an 8.23% increase in compressive strength compared to unfilled FPU. Acoustic analysis showed that the addition of BC improved absorption, and modified BC enhanced absorption characteristics of FPU, reaching Class D with a 20 mm thickness. BC modified with APTMS further improved acoustic properties compared to the unfilled FPU sample (Class E), with 20% modification showing the best results. These composites present promising materials for sound absorption applications and address environmental issues related to eggshell waste.

## Introduction

In recent years, rapid advancements in modern industries and transportation have resulted in significant noise pollution, which has a notable adverse impact on the environment and personal health, such as stress, depression and even hypertension^1,2^. One crucial approach to mitigate noise pollution involves enhancing sound absorption efficiency through an increase in damping capacity and optimization of material pore structures^3^. The mechanism of sound wave dissipation inside pore materials involves three main steps: Firstly, within porous sound-absorbing materials, air molecules experience vibrational motion and frictional interactions with the porous structure, precipitating the conversion of sound energy into thermal energy, subsequently dissipating it. Secondly, when longitudinal sound waves traverse these porous substrates, the air enclosed within the pores undergoes cyclic compression and decompression, entailing energy expenditure throughout the dynamic energy transformation process. Thirdly, sound energy converts into mechanical and thermal forms through the resonant behavior of the pore walls^2,4^. Constructing porous sound absorbers following these principles requires compliance with two essential criteria. Firstly, the constituent materials must demonstrate a significant presence of pores, including cavities, channels, or interstices. Secondly, these pores should possess suitable dimensions and interconnectivity to effectively facilitate sound waves' dissipation^5,6^.

Common examples of porous absorbers are fibrous materials like glass wool, rock wool, fiberglass, and foams^7,8^. Among various foam materials, flexible polyurethane (FPU) foam stands out as a widely utilized sound absorber due to its versatility, high elasticity, lightweight nature, and highly interconnected pores^9^. FPU comprises two main segments: the soft segment imparts elasticity, while the hard segment provides foam strength^10^. The production of FPU involves a reaction between polyol (R-(OH)_n≥2_) and isocyanate (R-(NCO)_n≥2_) in the presence of a catalyst, surfactant, and blowing agent, resulting in cavities with interconnected pore structures^11^. By adjusting the quantities of catalysts, stabilizer, and blowing agents, the cavity size, pore architecture of FPU can be meticulously controlled^3,12,13^.

Furthermore, modifying the FPU is crucial for improving its thermal, mechanical performance and acoustic properties. One of the most employed approaches involves adding fillers during foam preparation^14^. The filler’s type, size, dispersion, and concentration are crucial factors in obtaining a stable foam structure. Achieving optimal performance in FPU foam necessitates carefully balancing the amount of filler added during its preparation to prevent aggregation and ensure effective dispersion while avoiding negative impacts on foam quality and uniformity^15^. Additionally, ensuring interfacial compatibility between the filler surface and the FPU matrix is a key parameter for enhancing its physical, mechanical and acoustic properties^16^. The current research emphasizes rendering FPU production more environmentally friendly through the utilization of eco-friendly raw materials, the integration of renewable resources, and the effective management of waste in industrial processes. There is a growing need to develop low-cost materials by producing PU composites based on natural fillers. Numerous scholars have highlighted the successful incorporation of natural fillers into the PU matrix. This integration aims to produce cost-effective foam with exceptional physical and mechanical properties. These natural fillers encompass a variety of materials, including rich husk^17^, cellulose^18^, feather fiber^19^, eggshell^20^, chitosan^10^, walnut shells^21^, wood fiber^22^, sugarcane bagasse^23^ and nanoclays^24^.

The increasing use of eggs in our daily lives contributes to the production of eggshells, which are recognized as a significant solid waste byproduct in the food sector. The eggshell waste is often disposed of in landfills without proper treatment, leading to significant environmental concerns^25,26^. These abandoned eggshells contain around 96% calcium carbonate, 1% magnesium carbonate, and small amounts of organic content^15,27^, making them highly valuable and suitable for use as a natural filler in polymer materials. Hence, it is crucial to prioritize upcycling techniques for eggshell waste, not only to reduce the environmental consequences but also to utilize the valuable elements present in these discarded materials.

Biochar (BC) is a carbonaceous porous material that is produced through the process of pyrolysis. This process involves the conversion of biomass in a nitrogen-rich environment at temperatures ranging from below 450 °C to higher than 550 °C. The production temperature of the BC is a crucial factor that significantly influences its composition and properties^28,29^. It is considered an attractive filler that can be utilized as an alternative to expensive and complex prepared inorganic fillers, such as graphene and carbon nanotubes, during foam preparation for efficient sound absorption capability. This is due to its cost-efficiency, renewability, porous surface, and high surface area^30^. In addition, it contains various functional groups on its surface, including hydroxyl, carboxyl, and carbonyl groups^31^, which enhance physical crosslinking, H-type, with the polymer matrix^32^. Several research dedicated that the incorporation of BC to polymer enhanced its thermal and mechanical properties^33–38^. Furthermore, it was found that treated filler with silane coupling agents showed high filler dispersion and avoid filler agglomeration inside polymer matrix and consequently improve physical, mechanical and acoustic properties^16,39^.

Numerous investigations have elucidated the advantageous impact of filler incorporation on foam materials, particularly with respect to their mechanical performance and sound absorption characteristics. For instance, Kim et al.^40^ examined the effect of graphene on the sound absorption properties of FPU foam. Their findings indicated that introducing 0.2 wt % graphene into the FPU matrix resulted in a noteworthy 18.2% enhancement in sound absorption compared to unfilled FPU foam. In a separate study, the impact of different concentrations of MMT nanoclay (ranging from 0 to 0.7 wt. %) on the morphology and mechanical properties of FPU foam was studied^24^. Their results revealed that incorporating 0.3 wt. % MMT nanoclay led to a 32.47% reduction in cavity size and a 27.75% increase in compressive strength compared to FPU foam without MMT nanoclay. Verdejo et al.^41^ investigated the effect of carbon nanotubes on the sound absorption properties of FPU foam. Their outcomes showed that including 0.1 wt. % of carbon nanotubes led to a substantial 90% augmentation in the sound absorption coefficient. Maamoun et al.^11^ studied how halloysite clay affected the mechanical and acoustic performance of FPU foam. The results elucidated that the incorporation of 1 wt. % halloysite clay significantly enhanced the compression strength, achieving a remarkable 158.93% improvement relative to unfilled FPU foam. Additionally, this inclusion induced a shift in sound absorption towards lower frequency ranges. Furthermore, Choe et al.^16^ examined the effect of wood fibers and its treatment with (1–5 wt. %) (3-amonopropyl)triethoxysilane (APTMS) on the sound absorption of FPU foam. Their findings showcased that the incorporation of 1 wt. % wood fibers into the FPU foam matrix led to an impressive 86% enhancement in the sound absorption coefficient. Moreover, at 2 wt. % APTMS-wood treatment, the sound absorption showed a shift towards lower frequencies.

To the best of our knowledge, there is a lack of research in the literature regarding the effects of BC and its treatment using (3-aminopropyl)trimethoxysilane (APTMS) coupling agent on the thermal properties, compression behavior, and acoustic performance of FPU. This study is divided into three parts. The first part aims to investigate how different ratios of untreated BC (0.1, 0.3, 0.5, and 0.7 by weight) with a particle size of 23 nm affect the physical, thermal, compression, and acoustic properties of FPU foam. The second part focuses on treating the BC of particle size 23 nm with (3-aminopropyl)trimethoxysilane (APTMS) coupling agent at different weight percentages (10, 20, and 30 wt. %) to improve the dispersion of BC within the FPU matrix and optimize the modification. Lastly, two different particle sizes of treated BC (400 nm and 1800 nm), each with 20% APTMS, are prepared and integrated into FPU with 0.1 wt. % to examine the impact of filler particle size on physical properties, pore structure, compression, and acoustic performance.

## Experimental methods

### Materials

The FPU foams were prepared using Konix 1990 as a polyether-based polyol with 10 wt. % styrene-acrylonitrile solid particles provided by KPX chemicals, Korea, with an OH number of 41 mg KOH/g, the viscosity of 950 mPa.s, and an average molecular weight of 4100 g/mol. Lupranate T80, a toluene diisocyanate (TDI) blend consisting of 80% 2.4- and 20% 2.6-TDI isomers supplied by BASF Polyurethanes, Germany, with a molecular weight of 174.2 g/mol and an isocyanate content of 48.2 wt. %. Gelling and blowing catalysts (DABCO T-9 and DABCO 33-LV) were provided by Air Products, UK. The surfactant (DABCO DC5933) used as an emulsifier and cell stabilizer was purchased from Evonik, Germany. The chemical blowing agent was distilled water sourced from our laboratory. El Gomhorya Chemical Co., Egypt, provided N, N'-dimethylformamide (DMF). Chicken eggshells were obtained from daily household waste. (3-Aminopropyl)trimethoxysilane (APTMS. 97%) was obtained from Sigma-Aldrich. Acetone and ammonium hydroxide were purchased from El Gomhorya Chemical Co., Egypt.

### Preparation of untreated biochar (BC)

**Figure 1:**
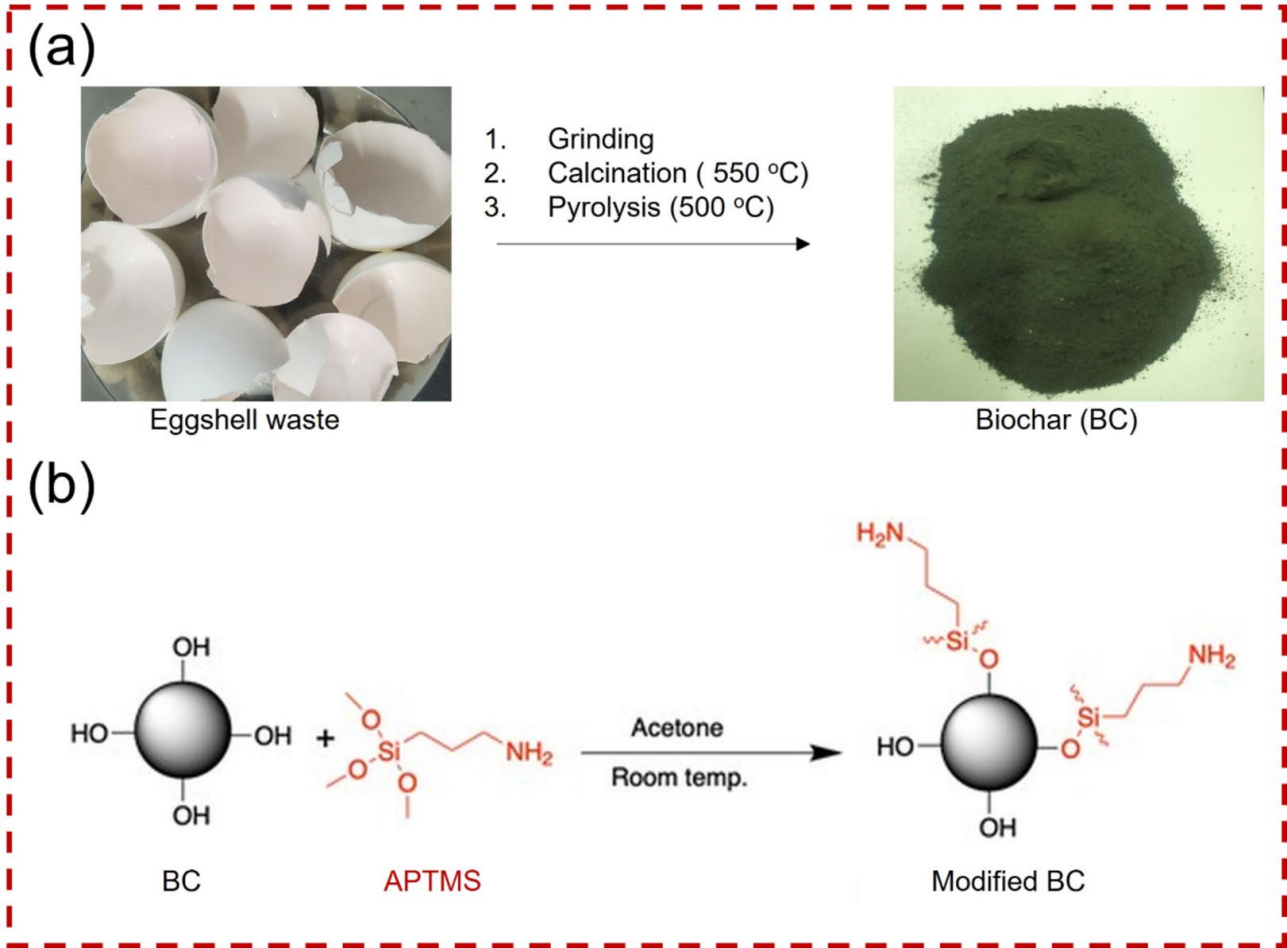
Illustration of (**a**) BC preparation from chicken eggshell waste, (**b**) modification of BC with APTMS.

In this study, BC was derived from chicken eggshell waste. First, we collected 50 g of chicken eggshell waste and washed it thoroughly to eliminate any pollutants. Afterwards, we allowed it to dry overnight, and the dried waste was then shredded. The crushed material underwent calcination in a muffle at a temperature of 550 °C for 1 h to eliminate any excess biological material and release the calcium carbonate. Subsequently, 10 g of the calcined powder were subjected to pyrolysis in a tube furnace set at 500 °C under a pure N_2_ flow of 50 sccm for up to 2 h with a heating rate of 10 °C/min. Finally, the obtained BC was cooled down using an N_2_ flow of 30 sccm to reach room temperature. The preparation steps of BC, the image of raw eggshells, and the obtained BC are visualized in Fig. 1a.

The received BC undergoes ball milling utilizing a Planetary Mill PULVERISETTE 5, outfitted with zirconium oxide grinding balls (10 mm diameter) under dry conditions. The grinding speed is seated at 800 rpm, and various grinding times of 5, 10, and 15 min were employed.

### Modification of BC with APTMS

The modified scheme of BC with APTMS is depicted in Fig. 1b. The modification procedure is outlined as follows: In a 150 mL bottle, 5 grams of BC with a particle size of 23 nm were dispersed in 100 mL of acetone using ultrasound at a frequency of 40 kHz for 15 min. Next, various amounts of APTMS were added to the dispersed BC. Specifically, 10, 20, and 30 wt. % of APTMS were used, corresponding to 0.5 g, 1 g, and 1.5 g, respectively. A catalytic amount of ammonium hydroxide (1–2 drops) was also added as a catalyst. The mixture was then subjected to an additional 15 min of sonication at 40 kHz to promote the reaction between the BC and APTMS. After sonication, the dispersed mixture was stirred at 800 rpm at room temperature for 3 h to complete the functionalization process^42^. Finally, the solvent was removed using a rotary evaporator, and the modified BC samples were dried for further characterization before being combined with FPU foam. The modified samples are denoted as S_BC10, S_BC20, and S_BC30, corresponding to the APTMS weight percentages of 10%, 20%, and 30%, respectively.

### Fabrication of FPU/untreated BC composites

**Figure 2:**
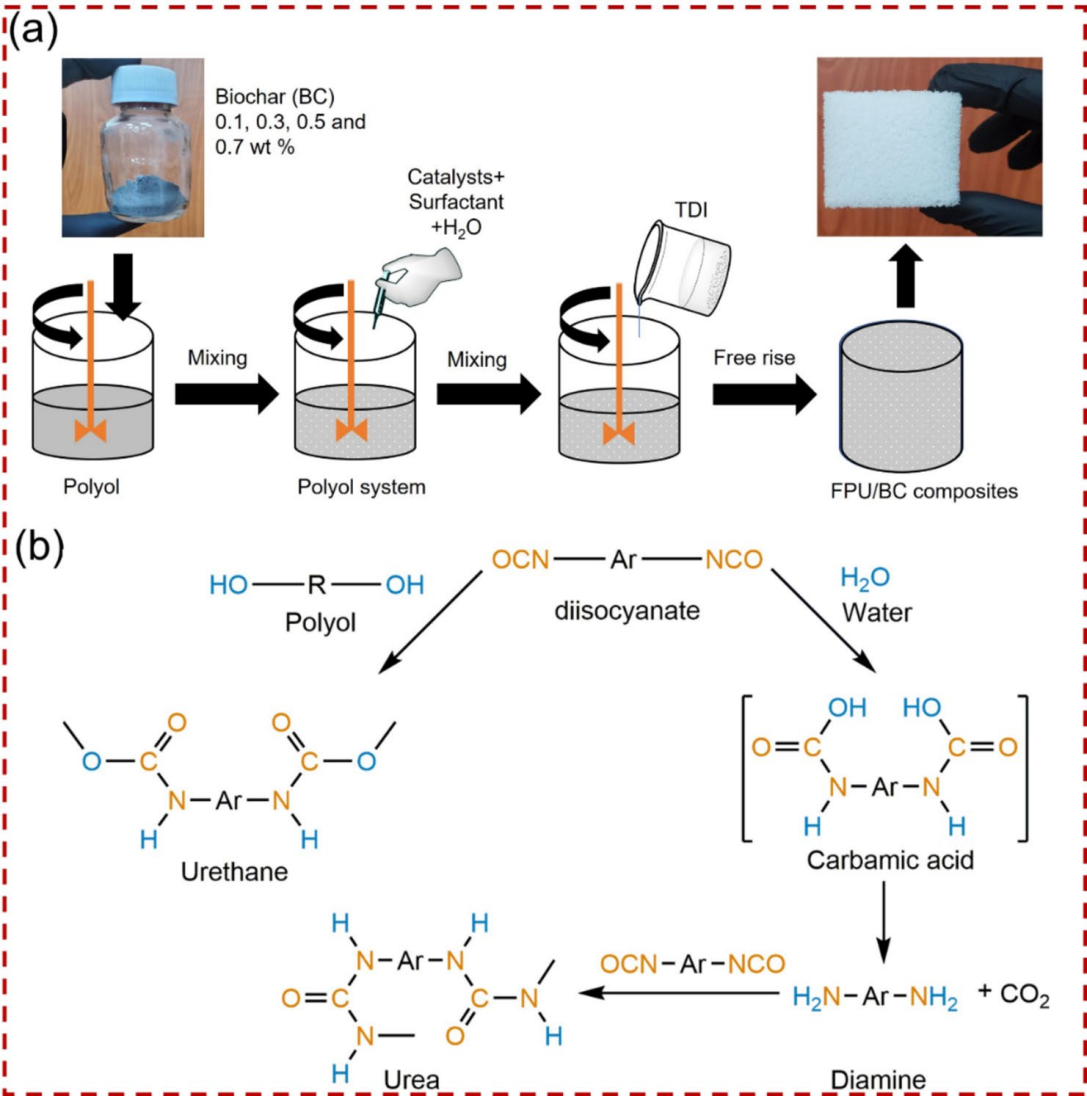
Schematic representation of (**a**) the reaction scheme for FPU preparation, and (**b**) the preparation of FPU/BC composite.

FPU samples were fabricated using a one-shot free-rise polymerization technique, following the procedure reported in the literature with minor modifications^24^. Initially, the polyol was dried for 24 h at 105 °C to eliminate any moisture. Subsequently, the polyol was allowed to cool under vacuum to room temperature before use. In a 500 mL polypropylene cup, the polyol, catalysts, stabilizer, and water-blowing agent were blended at 1500 rpm for 60 s. Following this, various ratios of untreated BC (0.1, 0.3, 0.5, and 0.7 wt. %) with a particle size of 23 nm were mixed into the polyol system until complete homogeneity was achieved. Finally, a stoichiometric amount of TDI was added to the polyol system and vigorously stirred at 3000 rpm for 6 s. The mixture was then quickly poured into a stainless-steel mould with dimensions of 200 × 200 × 200 mm^3^, and the samples were allowed to cure for 24 h before being cut for measurements. The preparation process for composites is depicted in the schematic representation shown in Fig. 2a. Table 1 depicts the typical formulation utilized in this study. The amount of TDI was calculated according to the following equation:

1 $$m_{TDI} = \left[ {\frac{{I_{{\left( {NCO} \right)}} }}{100}} \right]\left( {{\text{Eq}}_{{{\text{TDI}}}} } \right)\left[ {{ }\frac{{{\text{m}}_{{{\text{Polyol}}}} { }}}{{{\text{ Eq}}_{{{\text{Polyol}}}} }} + { }\frac{{{\text{m}}_{{{\text{H}}_{2} {\text{O}}}} { }}}{{{\text{ Eq}}_{{{\text{H}}_{2} {\text{O}}}} }}{ }} \right]{ }$$

where $m_{TDI}$ and ${\text{m}}_{{{\text{Polyol}}}}$ stand for the masses of TDI and polyol, while $I_{{\left( {NCO} \right)}}$ denote the isocyanate index. ${\text{m}}_{{{\text{H}}_{2} {\text{O}}}}$ represents the mass of water, and ${\text{Eq}}_{{{\text{TDI}}}}$, ${\text{Eq}}_{{{\text{Polyol}}}}$, and ${\text{Eq}}_{{{\text{H}}_{2} {\text{O}}}}$ correspond to the equivalent weights of TDI, polyol, and water, respectively.

**Table 1:** Typical formulations of unfilled FPU foam and FPU/BC composites.

Material	Formulations (Pphp*)
Polyol	100
Filler (Biochar)	0, 0.1, 0.3, 0.5, and 0.7 (wt. %)
DABCO T-9	0.17
DABCO 33-LV	0.2
DABCO DC5933	0.9
Distilled water	2.33
TDI	28.88

*Pphp, part per hundred part of polyol by weight.

The isocyanate index (NCO/OH) was adjusted to 1 in all formulations. The reaction scheme of FPU is presented in Fig. 2b. The obtained samples were labelled FPU/BC0.1, FPU/BC0.3, FPU/BC0.5, and FPU/BC0.7. The unfilled FPU foam was prepared with the same procedure without adding BC filler.

### Fabrication of FPU/treated BC composites

The silanized BC (S_BC), with varying percentages (10, 20, and 30 wt.%) of the APTMS coupling agent, was introduced into the FPU foam according to the procedure mentioned in the previous section. The prepared foams are denoted as FPU/S_BC10, FPU/S_BC20, and FPU/S_BC30. This modification aims to enhance the dispersion of BC within the FPU matrix and consequently improve foam properties. Additionally, silanized BC with 20 wt. % APTMS, of different sizes (400 nm and 1800 nm), were integrated into the FPU matrix and the obtained foam sample designated as FPU/S_BC400 and FPU/S_BC1800. To carefully examine the effect of modification and different particle sizes of treated BC, a constant weight ratio of 0.1 wt. % was employed, using the same formulation mentioned in Table 1.

### Characterization

Dynamic light scattering (DLS) was employed to investigate the particle size distribution of the prepared BC using the Zetasizer ZS (Malvern, UK). Acetone was used as the solvent, and the concentration of BC was kept constant at 0.5 wt/v%. The process involved dispersing BC in acetone for 10 min using an ultrasonic bath sonicator before the particle size measurement. Thermo Scientific Nicolet equipped with attenuated total reflection (ATR) to examine the chemical modification of the BC filler and the chemical structure of both the unfilled FPU and the prepared composite. The scanning rate covered a range from 400 to 4000/cm, with a resolution set at 4/cm. The morphologies of unmodified and modified BC, unfilled FPU, and FPU composites were analyzed using a Thermo Scientific Quattro S field emission scanning electron microscope (FESEM) equipped with energy-dispersive X-ray spectroscopy (EDX) to verify their elemental composition. The FPU samples were cut in the same direction of foam rise from the center of the foam block and affixed to carbon tape prior to imaging. The sizes of the cavities and pores in each foam sample were quantified using ImageJ software^43^. An average analysis was performed on three images for each foam sample, with approximately 100 cavities included in the analysis. The open porosity of the resulting composites was assessed using the following equation:

2 $$Open\; porosity \% = \frac{{V_{w} }}{{V_{t} }} \times 100$$

where $V_{t}$ represents the sample’s total volume, and $V_{w}$ signifies the volume filled by water within the sample. The formula for determining the water volume, $V_{w}$, is defined as $V_{w}$ = ($m_{wet}$−$m_{dry}$)/ρ, where mwet and mdry denote the wet and dry masses of the sample, respectively. The parameter ρ represents the density of water, with a specific value of ρ = 1000 kg/m^3^.

A solvent extraction method was employed to determine the gel fraction of the composites. Initially, the dry weight of the foam was measured. Subsequently, the foam was immersed in DMF for 3 days. Following this, the samples were placed in a vacuum oven at 70 °C for 24 h. The gel fraction was calculated using the following equation:

3 $${\text{Sol}}\;{\text{ fraction}} = \left[ {\frac{{W_{1} - W_{2} }}{{W_{1} }}} \right] \times 100\%$$

4 $$Gel \;fraction = 100\% - Sol \;fraction$$

where $W_{1}$ represents the initial sample weight, and $W_{2}$ corresponds to the sample’s weight after the drying process.

The ratio between the weight and volume of the sample was utilized to compute the apparent density of the prepared composites. An average of four samples was documented. The thermal stability of unfilled FPU and the selected composites were assessed using a LABSYS EVO TGA (SETARAM) apparatus within a controlled nitrogen atmosphere over a 25–800 °C temperature range at a heating rate of 10 °C/min. The weight of the samples used for the analysis was between 30 and 40 mg. According to the ASTM D-3574-17 test C standard, the compressive strength of the composite materials was evaluated using a universal testing machine (ZwickRoell/Z100, Germany) with a cell load of 1 kN. The testing process was carried out at a crosshead displacement rate of 50 mm/min and a deformation level of 50%. The sample dimensions were 50 × 50 × 40 mm, and the average of three samples was reported. The acoustic characteristics of the prepared FPU samples under consideration were investigated through their absorption coefficient. Samples were examined in the impedance tube apparatus where the normal incidence absorption coefficient (α_ni_) of a thin layer made of various materials can be measured^44^. According to ASTM E1050, a cylindrical shape with a diameter of 100 mm and thickness of 20 mm was used in this study. The values of α_ni_ were averaged over the three frequency ranges for easy comparison (low: less than 315 Hz, mid 315–1600 Hz, and high: above 1600 Hz)^45^. Furthermore, depending on the values of α_ni_, the following measures were computed:

- The Noise Reduction Coefficient (NRC)^46^, that is the arithmetic mean of the absorption coefficients at the four-octave band center frequencies: 250, 500, 1000, and 2000 Hz, rounded to the nearest multiple of 0.05.
- Sound Absorption Average (SAA)^46^, that is the mean of the 12 values of αni at one-third octave band center frequencies between 200 and 2500 Hz.
- The weighted sound absorption coefficient (α_w_) is calculated according to ISO 11654. According to ISO 11654, the following sound absorption ratings are given: ≥ 0.90 (A), 0.80–0.85 (B), 0.60–0.75 (C), 0.30–0.55 (D), 0.15–0.25 (E), and ≤ 0.10 (Not Classified).

The Origin Lab Statistics software^47^ was employed to conduct an Analysis of Variance and to assess the correlation between sound absorption coefficient (SAC) results and mechanical properties (MECH.). A linear regression model was created based on experimental variables using the least squares method. Additionally, the Pearson correlation coefficient was calculated to determine the relationship strength between SAC and MECH.

## Results and discussion

### Dynamic light scattering (DLS)

DLS analysis was utilized to determine the particle size of the prepared BC at different ball-milling times, specifically 5, 10, and 15 min. The particle size distribution curves are presented in Figs. S1–S3. The particle size is consistent with the ball-milling time, with longer times in the ball-mill resulting in smaller particle sizes. The particle sizes were 1800, 400, and 23 nm at 5, 10, and 15 min, respectively.

### Fourier transform infrared (FTIR) spectroscopy

The prepared BC particles were modified to enhance dispersion ability and improve the incorporation of the modified BC into the PU matrix. The chemical modification was confirmed using the FTIR technique. The FTIR spectra of untreated BC and modified BC with APTMS coupling agent are demonstrated in Fig. S4. The emergence of new bands at 1000 and 1125/cm is attributed to the Si–O–Si of APTMS^48^, confirming the successful incorporation of aminosilane in BC filler. The bands at 1400/cm and 876/cm are associated with C–O stretch and bending of calcite, respectively^49^. The band at 3422/cm is due to sorbed water. The amine group band overlaps with the adsorbed water band at 3400/cm, making it challenging to distinguish them. The aliphatic bands at 2930/cm increase in intensity after modification due to the introduction of a new propyl group to the system.

**Figure 3:**
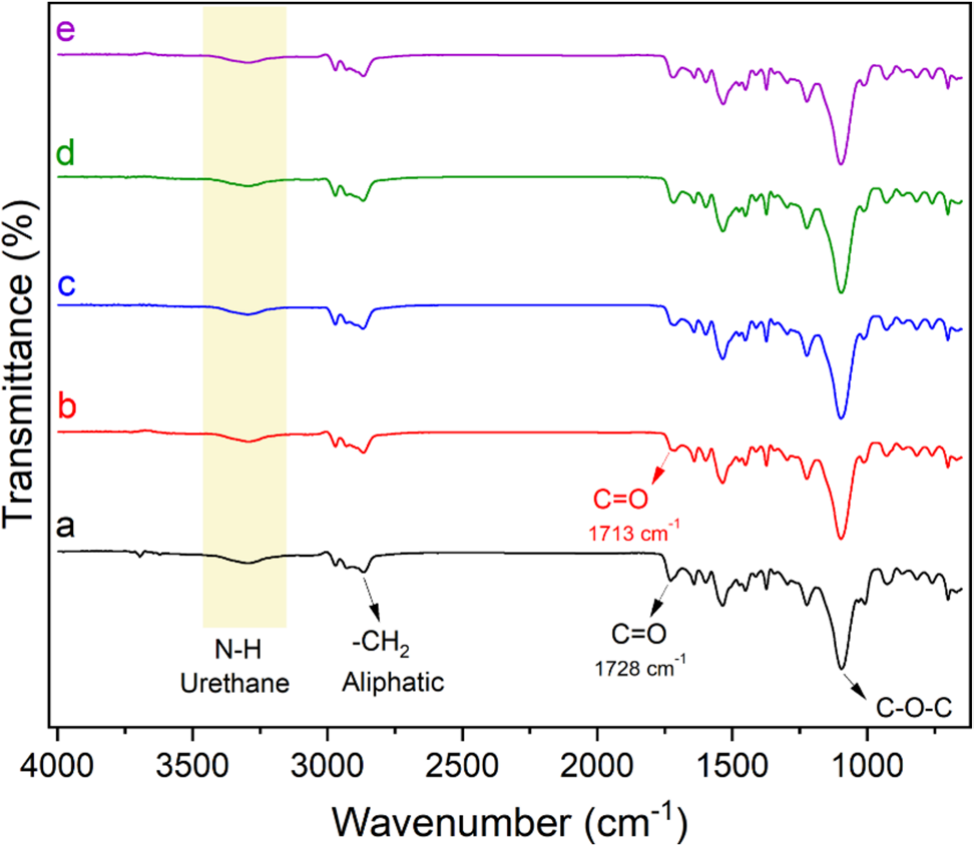
FTIR spectra of (**a**) unfilled FPU, (**b**) FPU/BC0.1, (**c**) FPU/S_BC20, (**d**) FPU/S_BC400, and (**e**) FPU/S_BC1800.

The chemical structure of the prepared composites was characterized using FTIR technique. Figure 3 depicts the typical FTIR spectrum of unfilled FPU foam and selected prepared composite materials. All well-defined bands derived from FPU bonds are present. The characteristic band at 3299/cm is attributed to the N–H stretching vibration^50^, indicating successful urethane formation^20^. The bands at 2970 and 2867/cm are associated with the asymmetric and symmetric C–H stretching of the aliphatic –CH_2_ group^10,51^. The absence of the band at 2275/cm indicates that all NCO groups reacted with the polyol^52^. Additionally, the physical interaction between urethanes and BC functional groups can be observed utilizing the FTIR spectrum. It is widely recognized that H-bonded carbonyl groups (–C=O) exhibit lower wavenumber infrared absorbance compared to free urethane carbonyls^17,52^. The unfilled FPU foam showed a band at 1728/cm, indicating unbonded (–C=O) of urethanes^50,53^. By adding BC filler, the band shifted to 1713/cm, indicating the formation of a physical interaction, H-bonded, between BC and urethane C=O groups. The bands at 1641 and 1095/cm are ascribed to the H-bonding of –C=O urea groups and the stretching vibration of C–O–C in the polyol^54,55^.

### Scanning electron microscopy (SEM)

**Figure 4:**
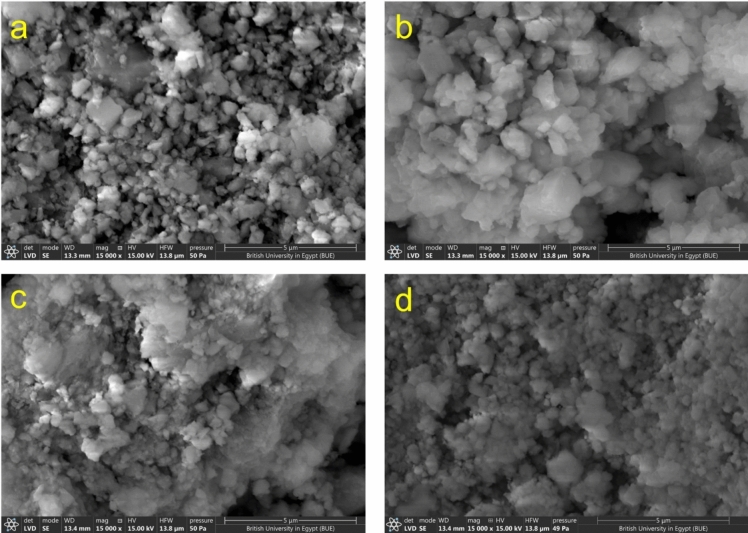
Typical SEM images of (**a**) untreated BC, (**b**) S_BC1800, (**c**) S_BC400, and (**d**) S_BC20.

The morphology of untreated BC and selected samples from modified BC at a magnification of 15,000× and working distance of 13.3 mm is illustrated in Fig. 4a–d. The images of untreated and modified BC reveal that the BC particles have an irregular surface and a granular-like structure (resembling calcite crystals)^56^. This is attributed to the calcination process at 550 °C that occurred before pyrolysis. Additionally, the EDX analysis confirmed the modification of BC with the APTMS coupling agent. To accurately quantify the composition of the elements, two random spots were selected, and the average values were recorded. Table S1compares the elemental composition of untreated BC and modified BC. The results show that untreated BC contains different ratios of calcium (Ca), carbon (C), and oxygen (O), attributed to calcite. In contrast, modified BC samples (S_BC20, S_BC400, and S_BC1800) have two new elements, nitrogen (N) and silicon (Si), with varying ratios, indicating the successful incorporation of APTMS moieties on the BC surface.

**Figure 5:**
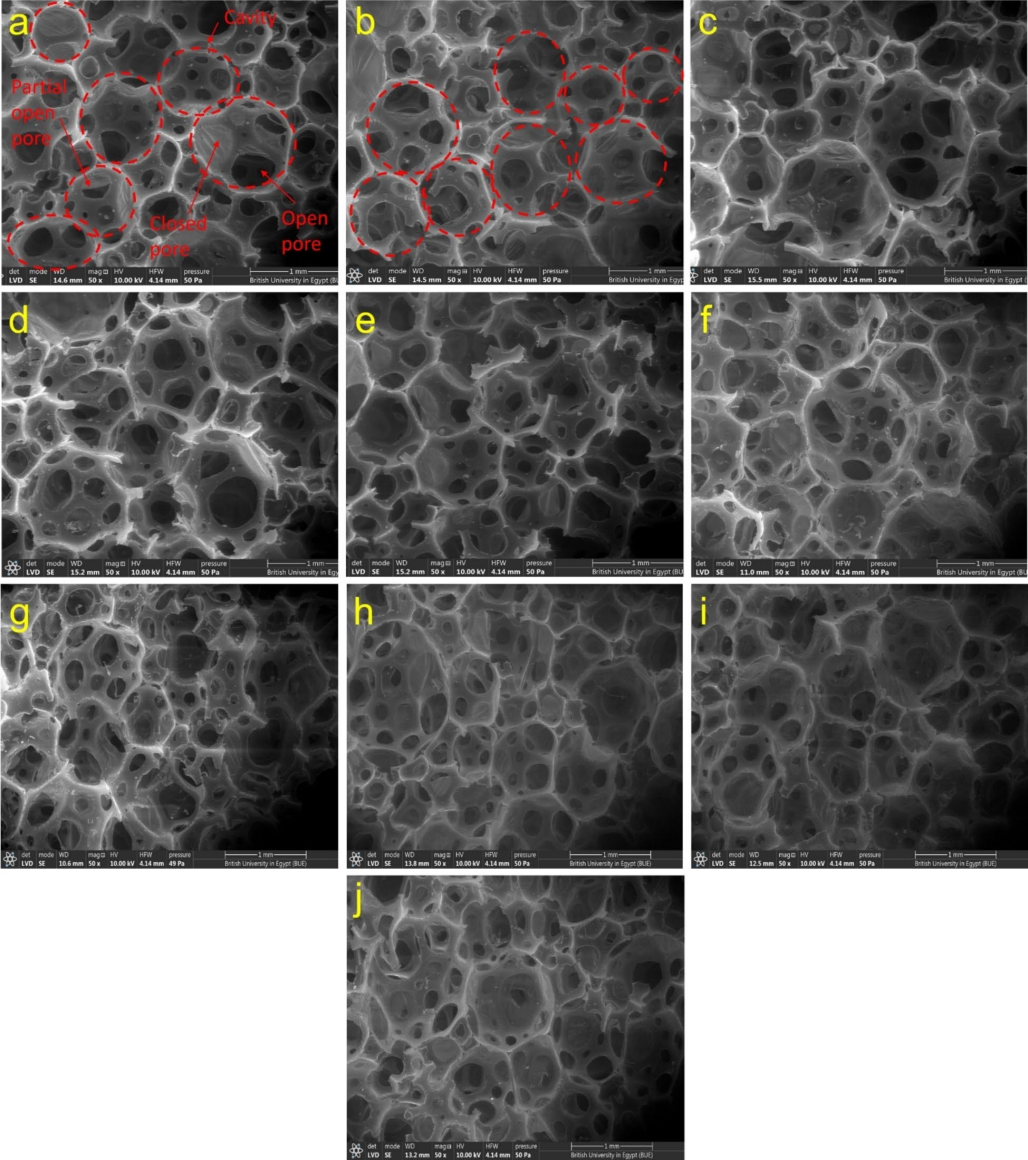
FESEM micrographs of (**a**) unfilled FPU, (**b**) FPU/BC0.1, (**c**) FPU/BC0.3, (d) FPU/BC0.5, (**e**) FPU/BC0.7, (**f**) FPU/S_BC10, (**g**) FPU/S_BC20, (**h**) FPU/S_BC30, (i) FPU/S_BC400, and (**j**) FPU/S_BC1800.

The morphology and pore architecture of the prepared foams play a crucial role in the efficient dissipation of sound waves^57,58^. Figure 5a–j displays the SEM micrographs of the fabricated foams. Generally, all micrographs contain oval cavities with three types of pores: closed, partially open, and completely open, depending on the thickness of the cavity walls and the PU gas drainage flow rate. The mechanism of cavity and pore formation is as follows: Closed pores develop when the polymerization reaction is completed prior to the rupture of the cavity walls; in this case, the walls of cavities are strong enough to withstand the gas pressure. Conversely, open pores arise due to the reduced strength of the walls and a high flow rate of drainage within the thin cavity walls. On the other hand, partial open pores occur when the wall strength is elevated and the drain flow rate is low; in this case, the pores may be neither fully closed nor completely open^59^

The average values of pore and cavity diameters are summarized in Table 2. The pore size distribution for the obtained foams is illustrated in Fig. S5. It is evident that all FPU composites exhibited lower cavity diameters than unfilled FPU foam, possibly due to the filler acting as nucleation sites, supporting cavity formation. Additionally, the diameter values depend on the dispersion of filler particles within the polymer matrix. For instance, the inclusion of 0.1 wt. % of unmodified BC resulted in lower values of 0.738 and 0.183 mm for cavity and pore diameter, respectively, indicating well-dispersed BC particles within the FPU matrix. However, when the BC content exceeds 0.1 wt. %, the cavity and pore sizes of FPU/BC composites begin to increase compared to FPU/BC0.1 sample. This could be attributed to a higher concentration of BC, leading to the agglomeration of BC particles, thereby destabilizing the cellular structure of the foam and resulting in larger cavities. Moreover, the impact of different wt. % of modified BC with APTMS on the cavity and pore diameters of FPU revealed no consistent trend. Remarkably, the highest values for both cavity and pore diameters were 0.922 and 0.305 mm, respectively, observed in FPU/S_BC1800, possibly due to the large particle size disturbing the nucleation process and forming large and irregular cavities.

**Table 2:** Values of cavity diameter, pore diameter, gel fraction and apparent density of unfilled FPU, FPU/Untreated BC (23 nm) composites, FPU/S_BC (10, 20, and 30 wt. % APTMS) composites and FPU/S_BC with different particle sizes (400 and 1800 nm).

Sample code	Cavity diameter	Pore diameter	Gel fraction	Apparent density
	(mm)	(mm)	(%)	(Kg/m^3^)
Unfilled FPU	1.008 ± 0.204	0.266 ± 0.111	90.54	34.96 ± 0.065
FPU/BC0.1	0.738 ± 0.125	0.183 ± 0.081	92.27	36.47 ± 0.082
FPU/BC0.3	0.873 ± 0.286	0.287 ± 0.092	91.10	36.12 ± 0.127
FPU/BC0.5	0.852 ± 0.169	0.223 ± 0.087	90.93	35.63 ± 0.017
FPU/BC0.7	0.849 ± 0.153	0.261 ± 0.103	90.24	34.80 ± 0.025
FPU/S_BC10	0.804 ± 0.145	0.265 ± 0.265	92.77	36.87 ± 0.064
FPU/S_BC20	0.851 ± 0.262	0.272 ± 0.136	93.76	37.70 ± 0.040
FPU/S_BC30	0.866 ± 0.236	0.247 ± 0.117	88.93	37.26 ± 0.028
FPU/S_BC400	0.818 ± 0.235	0.245 ± 0.075	91.78	36.88 ± 0.053
FPU/S_BC1800	0.922 ± 0.198	0.305 ± 0.091	89.34	35.94 ± 0.027

### Open porosity

**Figure 6:**
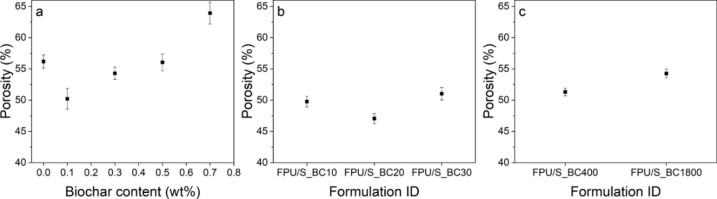
Open porosity of (**a**) unfilled FPU and FPU/untreated BC composites, (**b**) FPU/modified BC composites, and (**c**) FPU filled with modified BC of different particle sizes.

The relationship between open porosity and sound absorption coefficients is significant because open porous flow paths facilitate multiple sound wave collisions^16^. Figure 6a–c illustrates open porosity values for both unfilled FPU and FPU composites. It is evident that open porosity decreases with up to 0.1 wt. % of unmodified BC content but increases beyond this point, as depicted in Fig. 6a. This could be attributed to well-dispersed BC filler at this limit, which resulted in thicker walls and reduced drainage flow, consequently lowering the porosity. A similar trend is observed with modified BC, showcasing minimal porosity at 20% modified BC (0.1 wt. %), as seen in Fig. 6b. Moreover, introducing different particle sizes, 400 and 1800 nm, from modified BC impacts open porosity, as demonstrated in Fig. 6c, with FPU/S_BC1800 displaying higher porosity than FPU/S_BC400. This is believed to occur due to larger particle sizes disrupting cell formation and leading to thin cavity walls unable to withstand gas pressure.

### Gel fraction

The gel fraction, a crucial parameter for understanding the cross-linking in polymer composites, was measured. The gel fraction of unfilled FPU foam was 90.54%, which increased to 92.27% with the addition of 0.1 wt. % untreated BC (23 nm), as shown in Table 2. This increase was attributed to the high surface area of untreated BC, enhancing its adhesion to the polymer matrix and reducing sorption, and the possible reaction of the surface hydroxyl groups of untreated BC with isocyanate to increase the cross-linking. However, beyond 0.1 wt. %, the gel fraction decreased, possibly due to excess BC causing agglomeration. Furthermore, incorporating modified BC with an APTMS coupling agent increased the gel fraction, with a peak at 20% modification. However, the gel fraction deceased beyond this point. Additionally, the impact of treated BC particle size on the gel fraction was investigated, showing a decrease in the gel fraction as the particle size increased. This reduction was attributed to the lower surface area of larger particles, reducing the possibility of cross-linking with the FPU matrix and, consequently, the gel fraction.

### Apparent density

The density of flexible polyurethane (FPU) foam, mainly influenced by additives such as filler, is essential in measuring foam comfort and support. The density of FPU is directly proportional to its compressive properties. The densities of the obtained foams are listed in Table 2. Adding 0.1 wt. % of untreated BC filler to FPU foam increases foam density by 4.32% compared to unfilled FPU foam. However, beyond 0.1 wt. %, the density declines due to poor dispersion of the BC filler, which promotes cell rupture and more interconnected open pores, thus reducing the mass of the samples and consequently lowering the foam density. The influence of modified BC with different percentages of APTMS coupling agent on the density of FPU foam was also investigated. Adding 0.1 wt. % of S-BC10 and S-BC20 improved density compared to 0.1 wt. % of untreated BC and unfilled FPU foam. This improvement is attributed to the modification of BC with APTMS, which enhances the dispersion and compatibility of BC within the FPU matrix and produces a more crosslinking structure, thereby increasing density. However, beyond 20% modification, the density slightly decreased due to excessive modification with APTMS^16,39^, which facilitates particle agglomeration and poor dispersion of BC inside the FPU matrix, resulting in decreased density. The effect of BC particle size on foam density was also studied. It was observed that adding 0.1 wt. % of S-BC20 with 1800 nm particles resulted in a lower density than 0.1 wt. % of S-BC20 with 400 nm particles. This is due to the tendency of larger particles to aggregate, resulting in an inhomogeneous dispersion within the FPU matrix. Consequently, it is essential to adjust the quantity, size, and percentage of alteration to achieve the desired foam structure with optimal cellular morphology.

### Thermogravimetric analysis (TGA)

The thermal degradation behaviour of untreated BC and the modified BC sample is demonstrated in Fig. S6. The untreated BC exhibited a major decomposition step between 600 and 800 °C, indicating the decomposition of CaCO_3_^60^. On the other hand, for the S_BC20 sample, the decomposition temperature below 200 °C is attributed to moisture removal, while the decomposition between 400 and 800 °C is assigned to the decomposition of APTMS moieties^61^.

**Figure 7:**
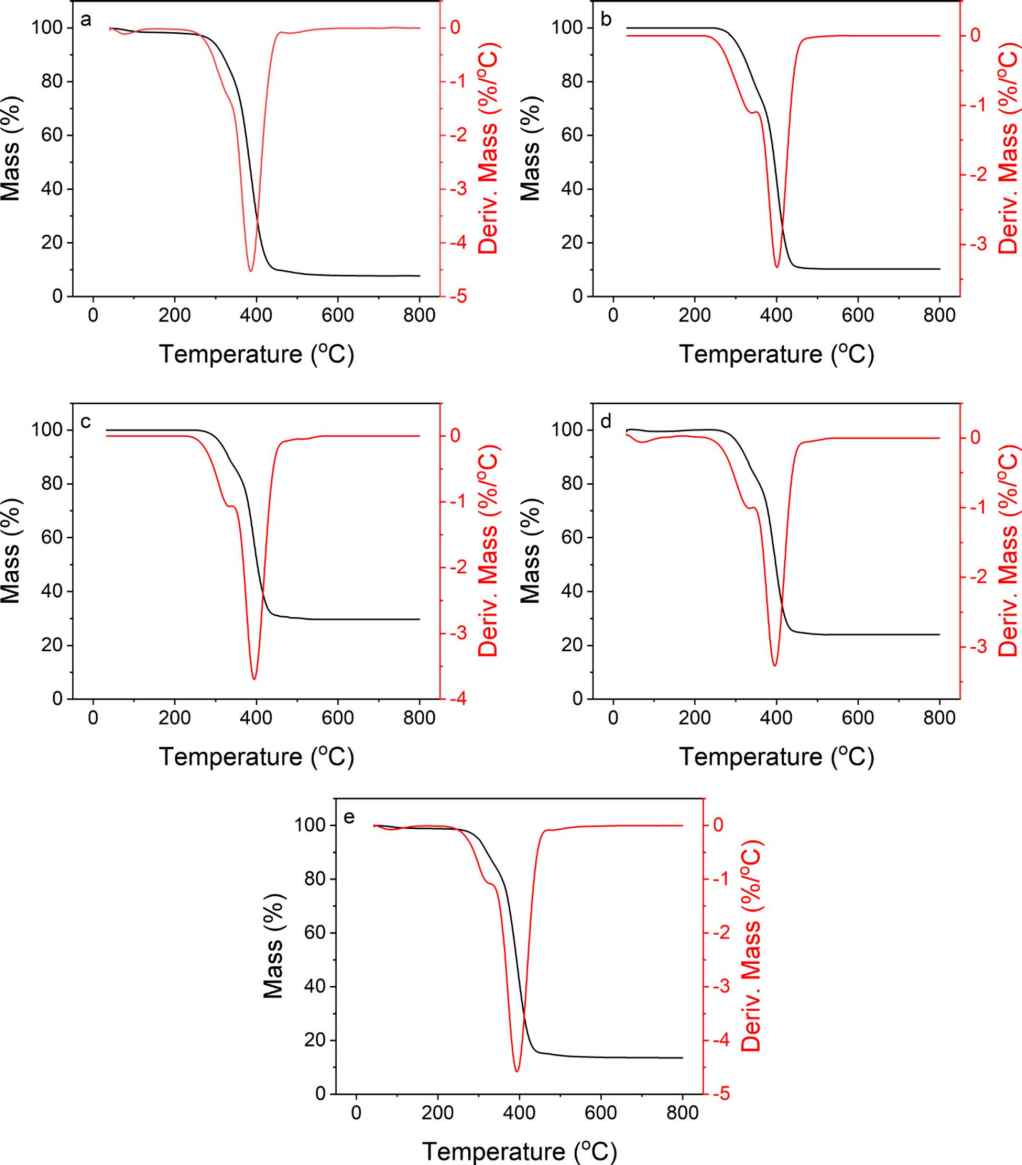
TGA/DTG plots of (**a**) unfilled FPU, (**b**) FPU/BC0.1, (**c**) FPU/S_BC20%, (**d**) FPU/S_BC400, and (**e**) FPU/S_BC1800.

Figure 7a–e presents TGA/DTG plots the thermal characteristics of unfilled FPU and selected composite materials. The plots emphasize the notable influence of untreated and treated BC on enhancing the thermal stability of FPU foam. Two distinct thermal decomposition phases were observed: the initial phase (250–350 °C) corresponding to the decomposition of urethane hard segments and a rapid degradation stage (350–450 °C) attributed to the decomposition of polyol soft segments^11^.

The detailed thermal decomposition characteristics, including the decomposition temperature at 5% and 50% mass loss (T_d5%_ and T_d50%_), and residual mass percentages at 800 °C, are presented in Table 3. For instance, unfilled FPU exhibited a T_d5%_ at 292.17 °C. The addition of 0.1 wt. % of untreated BC led to an increase in the T_d5%_ value to 299.15 °C, indicating enhanced thermal stability. Furthermore, the addition of 0.1 wt. % of silanized BC with 20% APTMS further increased thermal stability, with the T_d5%_ reaching 312.77 °C and 29.72% residue at 800 °C. This enhancement was attributed to the crosslinking effect of silane moieties, resulting in a more interconnected polymer network that delayed thermal decomposition. Moreover, comparing different particle sizes of silanized BC with 20% APTMS (400 nm and 1800 nm) revealed that including 400 nm particles increased the thermal stability of FPU foam compared to the larger 1800 nm particles. This improvement was primarily due to the larger surface area of the smaller particles, leading to enhanced interfacial adhesion with the FPU matrix and more effective dispersion throughout the foam, thereby delaying thermal degradation. The T_d50%_ values exhibited the same trend.

**Table 3:** TGA results of unfilled FPU and FPU composites.

Material	T_d5%_	T_d50%_	Residue at 800 °C
	^o^C	^o^C	%
Unfilled FPU	292.17	382.83	7.72
FPU/BC0.1	299.15	394.78	10.27
FPU/S_BC20	312.17	402.16	29.72
FPU/S_BC400	305.57	397.86	24.01
FPU/S_BC1800	299.46	392.60	13.54

### Compression strength

**Figure 8:**
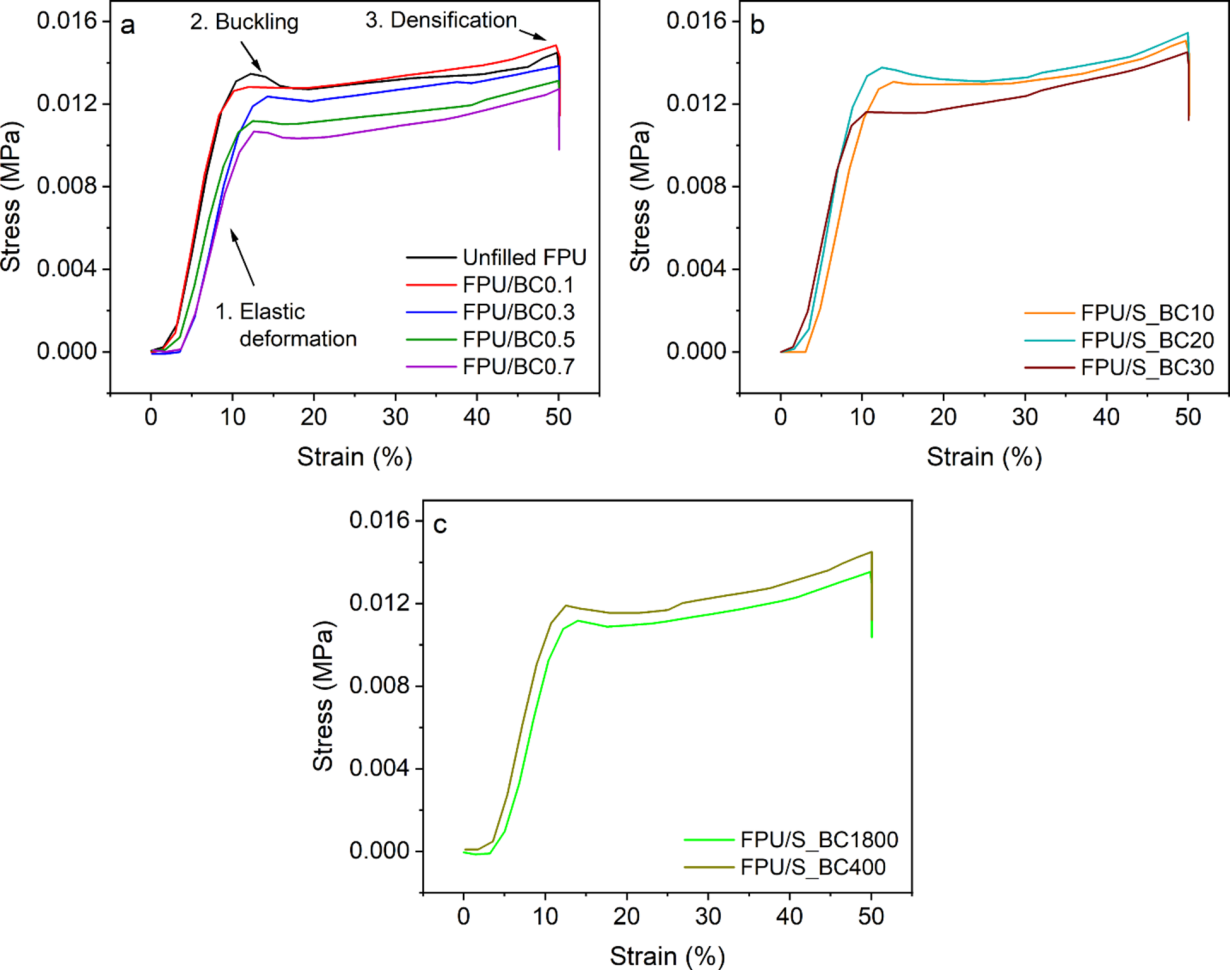
Typical compression stress–strain curve of (**a**) unfilled FPU and FPU/untreated BC composites with particle size 23 nm, (**b**) FPU/S_BC composites with varying APTMS ratios (10%, 20%, and 30 wt. %), and (**c**) FPU containing different particle sizes from S_BC (400 and 1800 nm).

Figure 8a–c presents stress–strain diagrams for unfilled FPU foam and the obtained FPU composites at 50% deformation. The diagrams display three distinct regions, each delineating its unique deformation mechanism. The initial region signifies elastic deformation, wherein the foam maintains structural integrity due to the inherent rigidity of its struts, thereby preventing collapse. The subsequent region depicts cell buckling, characterized by a length plateau in the central section. The third region denotes densification, which emerges under high-strain conditions as the polymer walls commence compressing against their adjacent counterparts^10^.

The results revealed that adding 0.1 wt. % of untreated BC increased compressive strength by 4.02% compared to unfilled FPU foam (Fig. 8a), which is attributed to improved dispersion and hydrogen bonding between the polymer matrix and BC. However, compressive strength decreased by 3.54%, 8.02%, and 12.65% for FPU/BC0.3, FPU/BC0.5, and FPU/BC0.7, respectively, compared with unfilled FPU foam. This decline may be attributed to BC particle agglomeration at higher concentrations, creating weak points in the foam structure that cannot withstand the applied load, resulting in reduced compressive strength. Additionally, decreased density and gel fraction contribute to this reduction. The same trend has been reported in previous studies^11,55,62^. Figure 8b displays the compression stress–strain curve for FPU filled with modified BC with different percentages of APTMS. The findings show that adding 0.1 wt. % of S_BC with 20% APTMS increases the compressive strength by 4.05% and 8.23% compared to FPU/BC0.1 and unfilled FPU foam, respectively. This enhancement results from BC modification with APTMS, which leads to improved dispersion and crosslinking with the FPU matrix through the NH_2_ groups. This, in turn, contributes to greater foam stiffness and, consequently, higher compression strength. However, when the modification exceeds 20%, the compression strength decreases. This reduction is attributed to an excessive silane percentage, which causes agglomeration due to hydrogen bonds forming between NH_2_ and OH groups on the surface of BC^16^.

Finally, we investigated how different particle sizes of S_BC20 (400 nm and 1800 nm) affect the compression strength of FPU, as shown in Fig. 8c. The results showed that incorporating S_BC20 with a particle size of 400 nm resulted in significantly higher compressive strength than the larger 1800 nm particles. This can be attributed to the smaller particles offering a larger surface area, which enhances their adhesion to the polymer matrix. Moreover, FPU containing S_BC20 particles measuring 23 nm exhibited greater compressive strength than foams with larger S_BC20 particles.

### Acoustic characteristics

**Figure 9:**
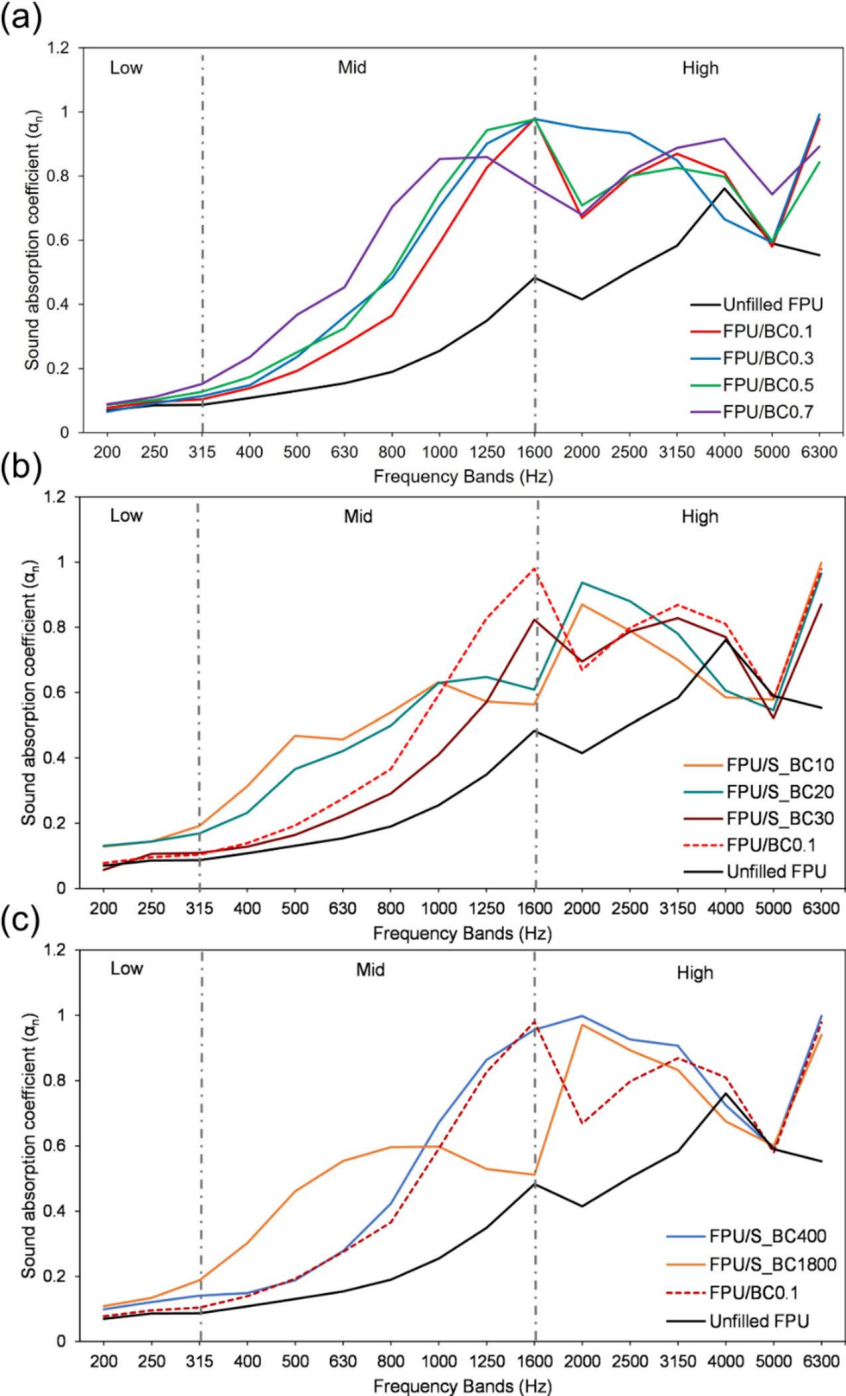
The normal incidence absorption coefficient (α_ni_) for (**a**) unfilled FPU and FPU/untreated BC composites, (**b**) FPU/S_BC composites. The two samples, unfilled FPU (the black line) and the FPU/BC0.1 (the dashed red line), are illustrated for comparison. (**c**) FPU containing different particle sizes from S_BC. Again, the two samples, unfilled FPU (the black line) and the FPU/BC0.1 (the dashed red line), are illustrated for comparison.

Table 4 illustrates the normal incidence absorption coefficient (α_ni_) per one-third octave center frequency for the three examined groups. The results of the first group, consisting of unfilled FPU and FPU/Untreated BC composites (five samples, 20 mm thick, 23 nm particle size), are depicted in Fig. 9a and reveal the following facts: In the low-frequency range, the values of α_ni_ range between 0.08 (for the unfilled FPU sample) and 0.12 (for the FPU/BC0.7 sample), with generally close values and no clear advantage for any sample. Moving to the mid-frequency range, the acoustic performance of the different samples becomes evident. The unfilled FPU exhibits the lowest performance, with an average normal absorption coefficient α_ni_ of 0.22. This value gradually increases with the percentage of added BC, reaching 0.55 for the FPU/BC0.7 composite. Excluding the FPU/BC0.7 sample, the values of α_ni_ gradually increase for the three samples (FPU/BC0.1, FPU/BC0.3, and FPU/BC0.5), indicating a progressive improvement in absorption characteristics up to the 1600 Hz band. In this mid-frequency range, it can be concluded that the best performance corresponds to the highest percentage of BC (0.5, 0.3, and then 0.1 wt. %), with the latter showing the lowest performance among the three samples, except for one exception at the 630 Hz band. The FPU/BC0.7 sample exhibits different behaviour, with notably higher absorption coefficients, but the three samples (FPU/BC0.1, FPU/BC0.3, and FPU/BC0.5) generally surpass it from the 1250 Hz band onwards. The absorption coefficient of the FPU/BC0.7 sample remains almost stable between the two bands at 1000 and 1250 Hz, followed by a sudden drop at the successive bands of 1600 and 2000 Hz, representing the threshold of the high-frequency range.

**Table 4:** Values of α_ni_ for the samples of the three groups, 20 mm thickness.

Sample	One-third octave center frequency (Hz)															
	Low		Mid								High					
	200	250	315	400	500	630	800	1000	1250	1600	2000	2500	3150	4000	5000	6300
Effect of untreated BC																
Unfilled FPU	0.07	0.09	0.09	0.11	0.13	0.15	0.19	0.26	0.35	0.48	0.42	0.50	0.58	**0.76**	0.59	0.55
FPU/BC0.1	0.08	0.10	0.10	0.14	0.19	0.28	0.37	0.59	0.83	**0.98**	0.67	0.80	0.87	0.81	0.58	**0.98**
FPU/BC0.3	0.07	0.09	0.12	0.15	0.24	0.36	0.48	0.71	0.90	0.98	0.95	0.93	0.85	0.67	0.59	**0.99**
FPU/BC0.5	0.09	0.10	0.13	0.17	0.25	0.33	0.50	0.75	0.94	**0.98**	0.71	0.80	0.83	0.80	0.60	0.84
FPU/BC0.7	0.09	0.11	0.15	0.24	0.37	0.45	0.70	0.85	0.86	0.77	0.68	0.81	0.89	**0.92**	0.74	0.89
Effect of modified BC with APTMS																
FPU/S_BC10	0.13	0.14	0.19	0.31	0.47	0.46	0.54	0.63	0.57	0.56	0.87	0.79	0.70	0.59	0.58	**1.00**
FPU/S_BC20	0.13	0.14	0.17	0.23	0.37	0.42	0.50	0.63	0.65	0.61	0.94	0.88	0.78	0.61	0.55	**0.97**
FPU/S_BC30	0.06	0.11	0.11	0.13	0.16	0.22	0.29	0.41	0.57	0.82	0.70	0.79	0.83	0.77	0.52	**0.87**
Effect of different particle size of S_BC20																
FPU/S_BC400	0.10	0.12	0.14	0.15	0.19	0.28	0.42	0.67	0.86	0.96	**1.00**	0.93	0.91	0.73	0.59	**1.00**
FPU/S_BC1800	0.11	0.13	0.19	0.30	0.46	0.55	0.60	0.60	0.53	0.51	**0.97**	0.89	0.83	0.68	0.60	0.94

Cells in bold show the maximum value(s) of α_ni_.

In the high-frequency range (above 1600 Hz), the behaviour of the unfilled FPU sample aligns with that of the other samples in this group, exhibiting a decrease in the normal absorption coefficient (α_ni_) at the 2000 Hz band, followed by an increase. However, the unfilled FPU sample showed a noticeable decline in acoustic performance compared to the other samples. In this range, the values of α_ni_ for the four other samples under discussion strongly overlap, making it challenging to distinguish the best through detailed interpretation. Nevertheless, the mean α_ni_ (see Table 5) indicates that the FPU/BC0.3 and FPU/BC0.7 samples perform the best and are very close, with mean values of 0.83 and 0.82, respectively. Following in descending order are the FPU/BC0.1 and FPU/BC0.5 samples, with comparable mean α_ni_ values of 0.78 and 0.76, respectively. The unfilled FPU sample holds the lowest mean α_ni_ of 0.57. Generally, α_ni_ values decrease at the 2000 Hz band for all samples, with a steeper decline in the FPU/BC0.1 and FPU/BC0.5 samples, followed by a gradual increase until the 4000 Hz band. At the 5000 Hz band, all samples exhibit a decrease in α_ni_ values, followed by a subsequent rise to different values. The FPU/BC0.3 sample demonstrates exceptional performance in this high-frequency range, with a gradual decrease in performance until the 5000 Hz band, succeeded by a sudden increase at the 6300 Hz band, setting it apart from the other samples.

**Table 5:** The mean α_ni_, NRC, SAA, α_w_, and Absorption Class of the Examined Samples.

Sample	α_ni_ (mean)			NRC^(a)^	SAA^(b)^	α_w_^(c)^	Class^(d)^
	Low	Mid	High				
Effect of untreated BC							
Unfilled FPU	0.10	0.22	0.57	0.20	0.24	0.25(H)	E
FPU/BC0.1	0.11	0.43	0.78	0.40	0.43	0.30(MH)	D
FPU/BC0.3	0.08	0.49	0.83	0.50	0.50	0.30(MH)	D
FPU/BC0.5	0.11	0.51	0.76	0.45	0.48	0.30(MH)	D
FPU/BC0.7	0.12	0.55	0.82	0.50	0.51	0.35(MH)	D
Effect of modified BC with APTMS							
FPU/S_BC10	0.18	0.47	0.75	0.55	0.47	0.40(H)	D
FPU/S_BC20	0.14	0.45	0.79	0.50	0.47	0.40(H)	D
FPU/S_BC30	0.13	0.34	0.75	0.35	0.36	0.25(H)	E
Effect of different particle size of S_BC20							
FPU/S_BC400	0.11	0.46	0.86	0.50	0.48	0.30(MH)	D
FPU/S_BC1800	0.13	0.47	0.82	0.55	0.49	0.45(H)	D

^a)^Noise Reduction Coefficient, the average of the absorption coefficient at the four centres 250, 500, 1000, and 2000 Hz. The result is rounded in increments of 0.05, see^4^.

^b)^Sound Absorption Average, the average of the absorption coefficients for the twelve one-third octave bands from 200 to 2500 Hz, see^4^.

^c)^Rated sound absorption, see^5^.

^d)^Rating of sound absorption, ≥ 0.90 (A), 0.80–0.85 (B), 0.60–0.75 (C), 0.30–0.55 (D), 0.15–0.25 (E), ≤ 0.10 (not classified), see^5^.

Generally, the values of the three measures SAA, NRC, and α_W_ (see Table 5) confirm the previous findings. In descending order, the SAA proves that the FPU/BC0.7 sample is the best (0.51), followed by the FPU/BC0.3 (0.50), FPU/BC0.5 (0.48), FPU/BC0.1 (0.43), and finally the unfilled FPU sample (0.24). The values of NRC come in a close context, where the two samples, FPU/BC0.7 and FPU/BC0.3, show the best results (NRC = 0.50), then in descending order, the samples FPU/BC0.5 (0.45), FPU/BC0.1 (0.40), and finally the unfilled FPU sample (0.20). Values of α_W_ confirm again that the FPU/BC0.7 is the best (0.35(MH), class D), followed by the three samples FPU/BC0.1, FPU/BC0.3, and FPU/BC0.5 with the same α_W_ (0.30(MH), class D) and finally the unfilled FPU sample (0.25(H), class E). Based on the previous findings, it can be concluded that adding BC evidently improves the acoustic performance of FPU foam. This improvement increases gradually by increasing the percentage of BC in the sample up to 0.70%, see Table 4. Table 4 lists the mean α_ni_ in the three frequency ranges (low, mid, and high) in addition to the values of NRC, SAA, α_W_, and the absorption class of the examined samples.

The second group (Fig. 9b) shows the effect of APTMS-modified BC on the acoustic performance of FPU foam. The results demonstrated that the modification enhanced the acoustic performance of FPU. The SAA and NRC values were higher for FPU/S_BC10 and FPU/S_BC20 than FPU/BC0.1, indicating improved performance. This is possibly due to the increased friction of sound waves with APTMS moieties, which facilitates the dissipation of sound waves. However, the FPU/BC30 sample exhibited the least favourable performance, with the lowest SAA and NRC values, as shown in Table 5. The two samples, FPU/S_BC10 and FPU/S_BC20, have almost identical results (SAA = 0.47, NRC = 0.55 and 0.50, respectively), with a slight increase in α_w_ noted for both, while a remarkable decrease occurs in the sample FPU/S_BC30. (α_w_ = 0.25(H) class E).

The last group examines the effect of BC particle size on the acoustic performance of FPU foam. This group includes two samples, 400 and 1800 nm (compared to the samples in the other two groups with a particle size of only 23 nm), both of which were added with 0.1 wt. %. Results clarified that the effect of the larger size is acoustically better up to the 800 Hz band, after which the effect of the smaller size (400 nm) is significantly acoustically superior in all bands above 800 Hz, see Fig. 9c, Tables 4 and 5. Nevertheless, the two measures, NRC and SAA, are too close (0.50 and 0.48 for the FPU/S_BC400, 0.55 and 0.49 for the 1800 nm). Values of αW are (0.30(MH) class D and 0.45(H) class D for the 400 and FPU/S_BC1800, respectively).

It worth recalls that the thickness of all samples under study is 20 mm. As Sabbagh and Elkhateeb^45^ showed, the standard absorption coefficient α_S_ “is directly proportional to the thickness of the polyurethane foam”; a remarkable improvement is expected by increasing the thickness of the samples to 40 mm or higher.

### Statistical analysis

A Pearson correlation study was performed to investigate the relationship between specified acoustical and mechanical parameters in this research, particularly examining how the sound absorption coefficient (SAC) correlates with mechanical properties (MECH). Regression analysis indicates a positive correlation between sound absorption coefficients (SACs) and mechanical properties (MECH.) which is significant (p < 0.05), across various composites, whether modified or unmodified, as shown in the Table 6. The table shows the values for Pearson’s correlation and p value, the correlation ranges from moderate to high in all cases, with values exceeding 0.5 and it is positive correlation in majority. The correlation between SACs is slightly stronger than between SACs and MECH. properties, or even among MECH. properties themselves, whether in modified or unmodified forms. Both the R-squared and adjusted R-squared values are very close, suggesting that the regression model is both suitable and effective. With a p-value less than 0.05, the results are statistically significant, confirming a strong correlation between sound and mechanical properties in the composites.

**Table 6:** Regression analysis of the SAC and MECH. properties studied in the current work.

		A	B	C	D	E	F	G	H	I	J	K	L	M	N	O	P	Q	R	S	T
SAC (unfilled FPU)	P C	1.0E+00	8.7E−01	7.8E−01	8.2E-01	8.3E−01	7.2E−01	7.6E−01	9.2E−01	8.2E−01	7.4E−01	7.0E−01	7.3E−01	8.1E−01	7.6E−01	7.8E−01	8.0E−01	7.0E−01	8.6E−01	8.2E−01	7.6E−01
	p	–	9.9E−06	3.3E−04	1.1E−04	5.8E−05	1.5E−03	6.5E−04	6.2E−07	1.1E−04	1.1E−03	2.3E−03	1.5E−03	1.5E−04	6.5E−04	3.8E−04	2.2E−04	2.4E−03	1.7E−04	1.1E−04	6.1E−04
SAC (FPU/BC0.1)	P C	8.7E−01	1.0E+00	9.6E−01	9.8E−01	9.2E−01	8.3E−01	8.8E−01	9.8E−01	9.7E−01	7.9E−01	8.1E−01	8.1E−01	9.0E−01	8.5E−01	8.7E−01	8.9E−01	8.1E−01	9.3E−01	9.1E−01	8.6E−01
	p	9.9E−06	–	1.6E−09	5.0E−11	4.6E−07	6.1E−05	5.8E−06	1.2E−10	7.2E−10	2.9E−04	1.7E−04	1.2E−04	2.6E−06	3.5E−05	1.1E−05	4.4E−06	1.5E−04	5.6E−06	1.2E−06	2.0E−05
SAC (FPU/BC0.3)	P C	7.8E−01	9.6E−01	1.0E+00	9.7E−01	9.1E−01	9.0E−01	9.5E−01	9.4E−01	9.9E−01	8.6E−01	8.5E−01	8.5E−01	9.2E−01	8.8E−01	9.0E−01	9.2E−01	8.5E−01	9.2E−01	9.3E−01	8.9E−01
	p	3.3E−04	1.6E−09	–	7.1E−10	1.0E−06	2.3E−06	2.5E−08	4.4E−08	2.7E−14	2.0E−05	3.7E−05	2.9E−05	4.1E−07	7.1E−06	1.8E−06	6.3E−07	2.9E−05	1.1E−05	1.9E−07	3.4E−06
SAC (FPU/BC0.5)	P C	8.2E−01	9.8E−01	9.7E−01	1.0E+00	9.5E−01	8.2E−01	8.7E−01	9.3E−01	9.6E−01	7.7E−01	8.6E−01	8.6E−01	9.3E−01	8.9E−01	9.2E−01	9.3E−01	8.7E−01	9.2E−01	9.4E−01	9.1E−01
	p	1.1E−04	5.0E−11	7.1E−10	–	1.5E−08	1.1E−04	1.1E−05	2.2E−07	4.5E−09	4.5E−04	1.7E−05	1.6E−05	1.2E−07	3.1E−06	5.9E−07	1.9E−07	1.5E−05	6.6E−06	3.9E−08	1.3E−06
SAC (FPU/BC0.7)	P C	8.3E−01	9.2E−01	9.1E−01	9.5E−01	1.0E+00	8.6E−01	8.8E−01	8.8E−01	8.9E−01	8.4E−01	9.4E−01	9.4E−01	9.8E−01	9.6E−01	9.7E−01	9.7E−01	9.4E−01	9.1E−01	9.8E−01	9.6E−01
	p	5.8E−05	4.6E−07	1.0E−06	1.5E−08	–	2.2E−05	8.5E−06	7.6E−06	3.2E−06	5.0E−05	8.7E−08	8.2E−08	1.3E−10	4.7E−09	4.2E−10	2.3E−10	1.0E−07	1.8E−05	5.3E−11	1.6E−09
SAC (FPU/S_BC10)	P C	7.2E−01	8.3E−01	9.0E−01	8.2E−01	8.6E−01	1.0E+00	9.8E−01	8.5E−01	8.9E−01	9.7E−01	8.5E−01	8.6E−01	8.8E−01	8.7E−01	8.7E−01	8.7E−01	8.5E−01	7.2E−01	8.7E−01	8.7E−01
	p	1.5E−03	6.1E−05	2.3E−06	1.1E−04	2.2E−05	–	2.2E−11	2.8E−05	4.7E−06	1.9E−10	2.7E−05	1.7E−05	8.8E−06	1.0E−05	1.1E−05	9.5E−06	2.5E−05	5.5E−03	1.2E−05	9.7E−06
SAC (FPU/S_BC20)	P C	7.6E−01	8.8E−01	9.5E−01	8.7E−01	8.8E−01	9.8E−01	1.0E+00	9.0E−01	9.5E−01	9.7E−01	8.4E−01	8.5E−01	8.9E−01	8.7E−01	8.8E−01	8.9E−01	8.4E−01	8.2E−01	8.9E−01	8.7E−01
	p	6.5E−04	5.8E−06	2.5E−08	1.1E−05	8.5E−06	2.2E−11	–	1.6E−06	3.5E−08	8.1E−10	5.1E−05	3.2E−05	3.5E−06	1.3E−05	7.3E−06	4.2E−06	4.3E−05	6.4E−04	3.2E−06	9.2E−06
SAC (FPU/S_BC30)	P C	9.2E−01	9.8E−01	9.4E−01	9.3E−01	8.8E−01	8.5E−01	9.0E−01	1.0E+00	9.6E−01	8.4E−01	7.6E−01	7.8E−01	8.6E−01	8.1E−01	8.3E−01	8.5E−01	7.7E−01	9.2E−01	8.7E−01	8.2E−01
	p	6.2E−07	1.2E−10	4.4E−08	2.2E−07	7.6E−06	2.8E−05	1.6E−06	–	2.3E−09	5.1E−05	6.1E−04	3.9E−04	1.7E−05	1.4E−04	6.2E−05	2.6E−05	5.5E−04	1.0E−05	1.0E−05	1.0E−04
SAC (FPU/S_BC400)	P C	8.2E−01	9.7E−01	9.9E−01	9.6E−01	8.9E−01	8.9E−01	9.5E−01	9.6E−01	1.0E+00	8.5E−01	8.0E−01	8.1E−01	9.0E−01	8.4E−01	8.7E−01	8.9E−01	8.1E−01	9.3E−01	9.1E−01	8.6E−01
	p	1.1E−04	7.2E−10	2.7E−14	4.5E−09	3.2E−06	4.7E−06	3.5E−08	2.3E−09	–	2.6E−05	1.9E−04	1.5E−04	2.7E−06	4.1E−05	1.2E−05	4.2E−06	1.6E−04	2.8E−06	1.1E−06	2.2E−05
SAC (FPU/S_BC1800)	P C	7.4E−01	7.9E−01	8.6E−01	7.7E−01	8.4E−01	9.7E−01	9.7E−01	8.4E−01	8.5E−01	1.0E+00	8.5E−01	8.6E−01	8.7E−01	8.7E−01	8.6E−01	8.7E−01	8.5E−01	6.8E−01	8.6E−01	8.7E−01
	p	1.1E−03	2.9E−04	2.0E−05	4.5E−04	5.0E−05	1.9E−10	8.1E−10	5.1E−05	2.6E−05	–	3.3E−05	1.8E−05	1.5E−05	1.3E−05	1.7E−05	1.4E−05	3.0E−05	1.1E−02	2.2E−05	1.5E−05
MECH (Unfilled FPU)	P C	7.0E−01	8.1E−01	8.5E−01	8.6E−01	9.4E−01	8.5E−01	8.4E−01	7.6E−01	8.0E−01	8.5E−01	1.0E+00	9.9E−01	9.7E−01	9.9E−01	9.6E−01	9.7E−01	1.0E+00	6.1E−01	9.4E−01	9.6E−01
	p	2.3E−03	1.7E−04	3.7E−05	1.7E−05	8.7E−08	2.7E−05	5.1E−05	6.1E−04	1.9E−04	3.3E−05	–	8.6E−97	4.6E−61	1.2E−78	4.2E−55	7.3E−61	1.1E−103	4.4E−11	4.9E−45	1.9E−54
MECH (FPU/BC0.1)	P C	7.3E−01	8.1E−01	8.5E−01	8.6E−01	9.4E−01	8.6E−01	8.5E−01	7.8E−01	8.1E−01	8.6E−01	9.9E−01	1.0E+00	9.8E−01	1.0E+00	9.8E−01	9.8E−01	1.0E+00	7.2E−01	9.6E−01	9.8E−01
	p	1.5E−03	1.2E−04	2.9E−05	1.6E−05	8.2E−08	1.7E−05	3.2E−05	3.9E−04	1.5E−04	1.8E−05	8.6E−97	–	1.3E−68	1.5E−101	1.3E−67	2.4E−71	3.9E−116	1.9E−16	2.0E−53	4.2E−68
MECH (FPU/BC0.3)	P C	8.1E−01	9.0E−01	9.2E−01	9.3E−01	9.8E−01	8.8E−01	8.9E−01	8.6E−01	9.0E−01	8.7E−01	9.7E−01	9.8E−01	1.0E+00	9.9E−01	9.9E−01	1.0E+00	9.8E−01	8.5E−01	9.9E−01	9.8E−01
	p	1.5E−04	2.6E−06	4.1E−07	1.2E−07	1.3E−10	8.8E−06	3.5E−06	1.7E−05	2.7E−06	1.5E−05	4.6E−61	1.3E−68	–	1.7E−85	3.6E−85	6.6E−113	4.4E−65	2.2E−27	6.7E−78	7.1E−74
MECH (FPU/BC0.5)	P C	7.6E−01	8.5E−01	8.8E−01	8.9E−01	9.6E−01	8.7E−01	8.7E−01	8.1E−01	8.4E−01	8.7E−01	9.9E−01	1.0E+00	9.9E−01	1.0E+00	9.9E−01	9.9E−01	9.9E−01	8.0E−01	9.8E−01	9.9E−01
	p	6.5E−04	3.5E−05	7.1E−06	3.1E−06	4.7E−09	1.0E−05	1.3E−05	1.4E−04	4.1E−05	1.3E−05	1.2E−78	1.5E−101	1.7E−85	–	2.4E−85	5.4E−93	4.9E−93	3.3E−22	2.8E−66	1.3E−82
MECH (FPU/BC0.7)	P C	7.8E−01	8.7E−01	9.0E−01	9.2E−01	9.7E−01	8.7E−01	8.8E−01	8.3E−01	8.7E−01	8.6E−01	9.6E−01	9.8E−01	9.9E−01	9.9E−01	1.0E+00	1.0E+00	9.7E−01	8.9E−01	9.9E−01	1.0E+00
	p	3.8E−04	1.1E−05	1.8E−06	5.9E−07	4.2E−10	1.1E−05	7.3E−06	6.2E−05	1.2E−05	1.7E−05	4.2E−55	1.3E−67	3.6E−85	2.4E−85	–	5.0E−102	7.4E−64	1.8E−33	8.7E−97	2.0E−121
MECH (FPU/S_BC10)	P C	8.0E−01	8.9E−01	9.2E−01	9.3E−01	9.7E−01	8.7E−01	8.9E−01	8.5E−01	8.9E−01	8.7E−01	9.7E−01	9.8E−01	1.0E+00	9.9E−01	1.0E+00	1.0E+00	9.8E−01	8.7E−01	9.9E−01	9.9E−01
	p	2.2E−04	4.4E−06	6.3E−07	1.9E−07	2.3E−10	9.5E−06	4.2E−06	2.6E−05	4.2E−06	1.4E−05	7.3E−61	2.4E−71	6.6E−113	5.4E−93	5.0E−102	–	2.0E−68	7.1E−30	5.6E−87	4.7E−87
MECH (FPU/S_BC20)	P C	7.0E−01	8.1E−01	8.5E−01	8.7E−01	9.4E−01	8.5E−01	8.4E−01	7.7E−01	8.1E−01	8.5E−01	1.0E+00	1.0E+00	9.8E−01	9.9E−01	9.7E−01	9.8E−01	1.0E+00	6.8E−01	9.5E−01	9.7E−01
	p	2.4E−03	1.5E−04	2.9E−05	1.5E−05	1.0E−07	2.5E−05	4.3E−05	5.5E−04	1.6E−04	3.0E−05	1.1E−103	3.9E−116	4.4E−65	4.9E−93	7.4E−64	2.0E−68	–	2.5E−14	8.5E−51	2.3E−64
MECH (FPU/S_BC30)	P C	8.6E−01	9.3E−01	9.2E−01	9.2E−01	9.1E−01	7.2E−01	8.2E−01	9.2E−01	9.3E−01	6.8E−01	6.1E−01	7.2E−01	8.5E−01	8.0E−01	8.9E−01	8.7E−01	6.8E−01	1.0E+00	9.3E−01	8.9E−01
	p	1.7E−04	5.6E−06	1.1E−05	6.6E−06	1.8E−05	5.5E−03	6.4E−04	1.0E−05	2.8E−06	1.1E−02	4.4E−11	1.9E−16	2.2E−27	3.3E−22	1.8E−33	7.1E−30	2.5E−14	–	1.2E−43	4.6E−33
MECH (FPU/S_BC400)	P C	8.2E−01	9.1E−01	9.3E−01	9.4E−01	9.8E−01	8.7E−01	8.9E−01	8.7E−01	9.1E−01	8.6E−01	9.4E−01	9.6E−01	9.9E−01	9.8E−01	9.9E−01	9.9E−01	9.5E−01	9.3E−01	1.0E+00	9.9E−01
	p	1.1E−04	1.2E−06	1.9E−07	3.9E−08	5.3E−11	1.2E−05	3.2E−06	1.0E−05	1.1E−06	2.2E−05	4.9E−45	2.0E−53	6.7E−78	2.8E−66	8.7E−97	5.6E−87	8.5E−51	1.2E−43	–	2.8E−85
MECH (FPU/S_BC1800)	P C	7.6E−01	8.6E−01	8.9E−01	9.1E−01	9.6E−01	8.7E−01	8.7E−01	8.2E−01	8.6E−01	8.7E−01	9.6E−01	9.8E−01	9.8E−01	9.9E−01	1.0E+00	9.9E−01	9.7E−01	8.9E−01	9.9E−01	1.0E+00
	p	6.1E−04	2.0E−05	3.4E−06	1.3E−06	1.6E−09	9.7E−06	9.2E−06	1.0E−04	2.2E−05	1.5E−05	1.9E−54	4.2E−68	7.1E−74	1.3E−82	2.0E−121	4.7E−87	2.3E−64	4.6E−33	2.8E-85	–

P C: Pearson Corr.; p: p-value; (p-value) < 0.05: significance.

A: SAC (Unfiled FPU); B: SAC (FPU/BC0.1); C: SAC (FPU/BC0.3); D: SAC (FPU/BC0.5); E: SAC (FPU/BC0.7); F: SAC (FPU/S_BC10); G: SAC (FPU/S_BC20); H: SAC (FPU/S_BC30); I: SAC (FPU/S_BC400); J: SAC (FPU/S_BC1800); K: MECH. (Unfiled FPU); L: MECH. (FPU/BC0.1); M: MECH. (FPU/BC0.3); N: MECH. (FPU/BC0.5); O: MECH. (FPU/BC0.7); P: MECH. (FPU/S_BC10); Q: MECH. (FPU/S_BC20); R: MECH. (FPU/S_BC30); S: MECH. (FPU/S_BC400); T: MECH. (FPU/S_BC1800).

## Conclusion

This study is pivotal in mitigating sound pollution and addressing environmental concerns associated with eggshell waste. The BC derived from eggshell waste was successfully prepared through pyrolysis at 500 °C. The influence of untreated BC, APTMS-modified BC, and different particle sizes of BC on the physical, thermal, compression performance, and acoustic properties of FPU foam was thoroughly investigated. The outcomes showed that FPU composites were successfully prepared, as confirmed by FTIR spectroscopy and EDX. All composites exhibited a decrease in cavity size compared to the unfilled FPU sample. The addition of BC up to 0.1 wt.% to FPU caused a decrease in porosity and an increase in density; beyond this ratio, a reverse trend was observed. Furthermore, APTMS-modified BC showed improvement in the characteristics of FPU foam compared to unfilled foam and FPU/untreated BC, which is attributed to the well-dispersion of modified BC within the FPU matrix. TGA results of the prepared composites rose compared to pristine foam, indicating the ability of BC to delay the decomposition of FPU foam. Compression strength increased with 0.1 wt. % untreated BC but decreased beyond this ratio. Modified BC at 20%, APTMS exhibited enhanced compressive strength. Moreover, acoustic measurements demonstrated that adding BC particles improved the acoustic performance of FPU foam, with the best performance corresponding to the highest percentage of BC (0.7 wt.%). The addition of APTMS-modified BC particles further enhanced the acoustic performance of FPU foam compared to the unfilled foam sample. Additionally, the larger particle size of BC negatively affected the acoustic performance of FPU compared to the smaller one. Thus, precise adjustments in filler quantity, modification percentage, and particle size are imperative to attain the desired properties for the intended application.

### Supplementary Information

Supplementary Information.

## Data Availability

All data generated or analyzed during this study are included in this article.
