# Supplementary material for: Improving the acoustic performance of flexible polyurethane foam using biochar modified by (3-aminopropyl)trimethoxysilane coupling agent
Source: Sci Rep. 2024 Aug 8;14:18382. doi: 10.1038/s41598-024-68039-w (PMC11310357; doi:10.1038/s41598-024-68039-w)
Supplement: Supplementary file 1 — Supplementary Information. [file 41598_2024_68039_MOESM1_ESM.docx]

**Supporting information**

Improving the Acoustic Performance of Flexible Polyurethane Foam Using Biochar Modified by (3-Aminopropyl)trimethoxysilane Coupling Agent

Ahmed Abdelhamid Maamoun^1,*^, Ramadan M. Abouomar^2,*^, Tarek M. El-Basheer^3^, Mostafa A. Azab^2^, ElSayed G. Zaki^2^, Shymaa M. Elsaeed^2^, Ahmed Elkhateeb^4^

^1^ Department of Engineering Physics and Mathematics, Chemistry Division, Faculty of Engineering, Ain Shams University, 1 EL-Sarayat Street - Abdo Basha Sq., Cairo, 11517, Egypt.

^2^ Egyptian Petroleum Research Institute, Nasr City, Cairo, 11727, Egypt.

^3^ Department of Acoustics, Mass, and Force Metrology Division, National Institute of Standards (NIS). El-Sadat Street, El-Haram, El-Giza, 12211, Egypt.

^4^ Department of Architecture, Faculty of Engineering, Ain Shams University, 1 EL-Sarayat

Street - Abdo Basha Sq., Cairo, 11517, Egypt.

*Corresponding author: Dr. Ahmed Abdelhamid Maamoun, Tel: +0201004898757

Email: [Ahmed.maamoun@eng.asu.edu.eg](mailto:Ahmed.maamoun@eng.asu.edu.eg)

*Corresponding author: Dr. Ramadan M. Abouomar, Tel: +0201063938974

Email: [rabouomar2016@gmail.com](mailto:rabouomar2016@gmail.com)

The file contains:

Table S1

Figures S1-S6

**Table S1** EDX data of untreated BC and selected samples from modified BC.

| Sample | Element (wt.%) | | | |  |
| --- | --- | --- | --- | --- | --- |
|  | Ca | C | N | O | Si |
| Untreated BC | 38.08 | 24.16 | - | 37.76 | - |
| S_BC20 | 38.37 | 11.56 | 18.25 | 29.27 | 2.55 |
| S_BC400 (20% APTMS) | 30.94 | 14.43 | 19.37 | 33.15 | 2.11 |
| S_BC1800 (20% APTMS) | 33.07 | 14.34 | 18.69 | 30.75 | 3.14 |


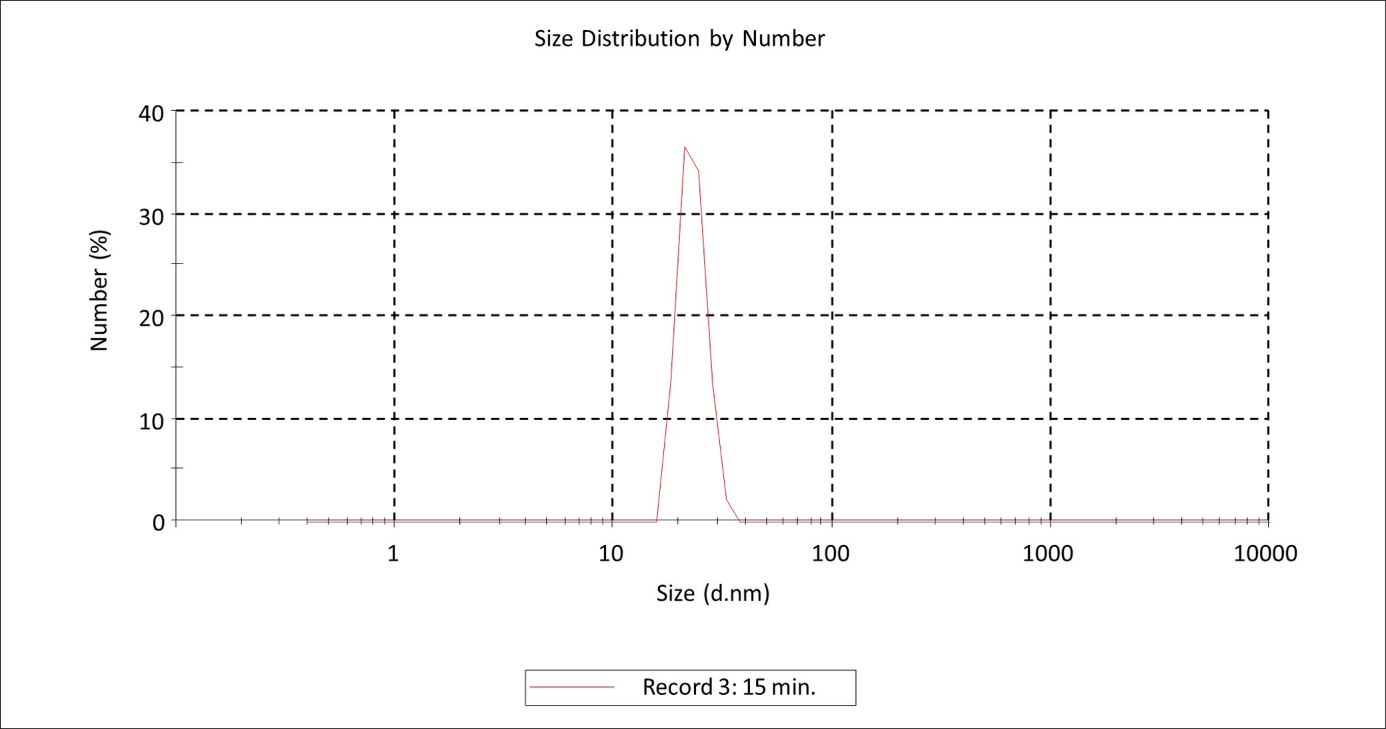


**Figure S1** Particle size distribution of BC after 15 minutes of ball-milling.


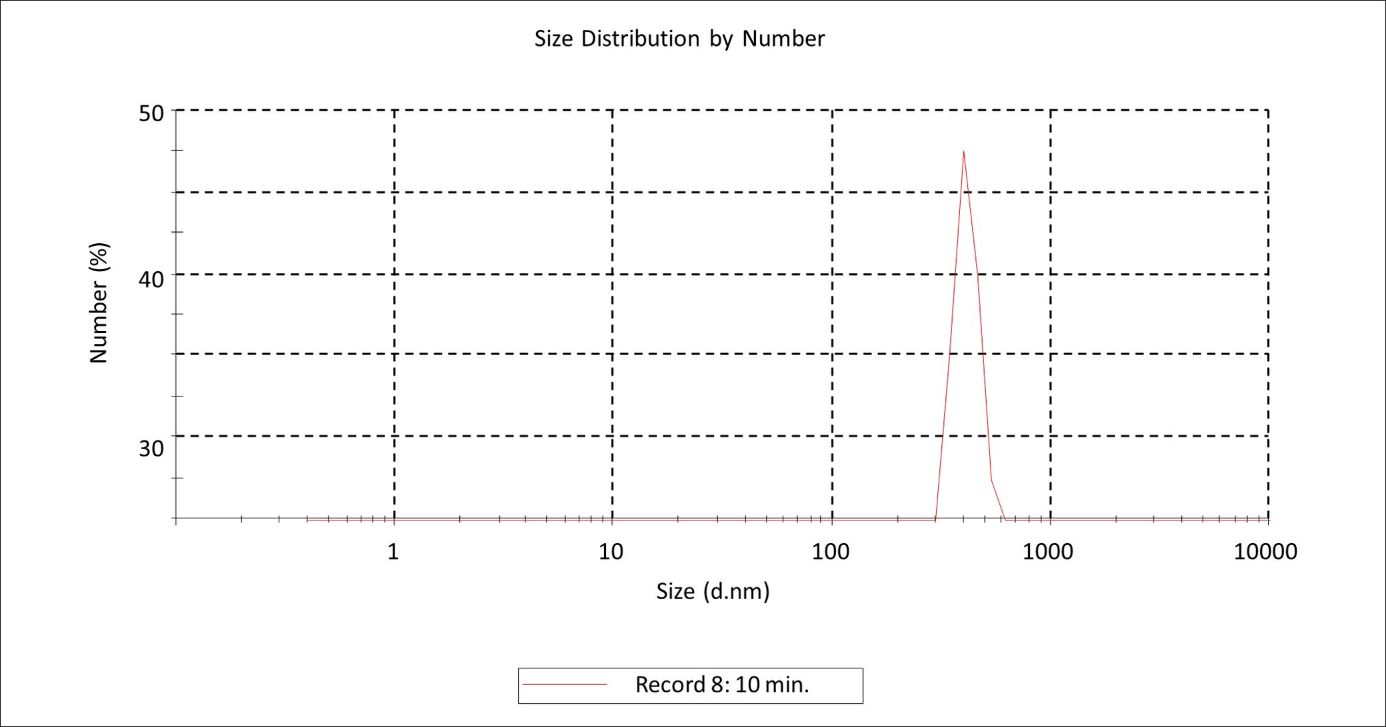


**Figure S2** Particle size distribution of BC after 10 minutes of ball-milling.


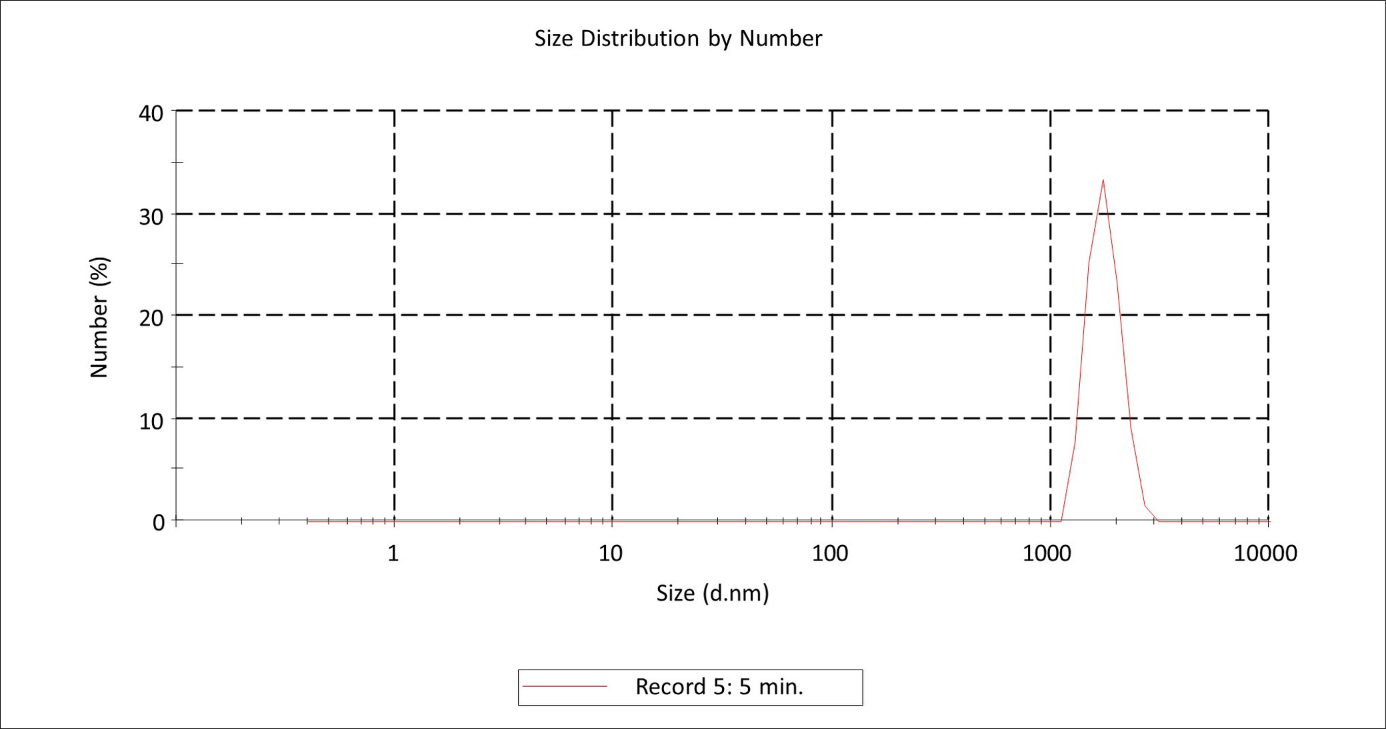


**Figure S3** Particle size distribution of BC after 5 minutes of ball-milling.


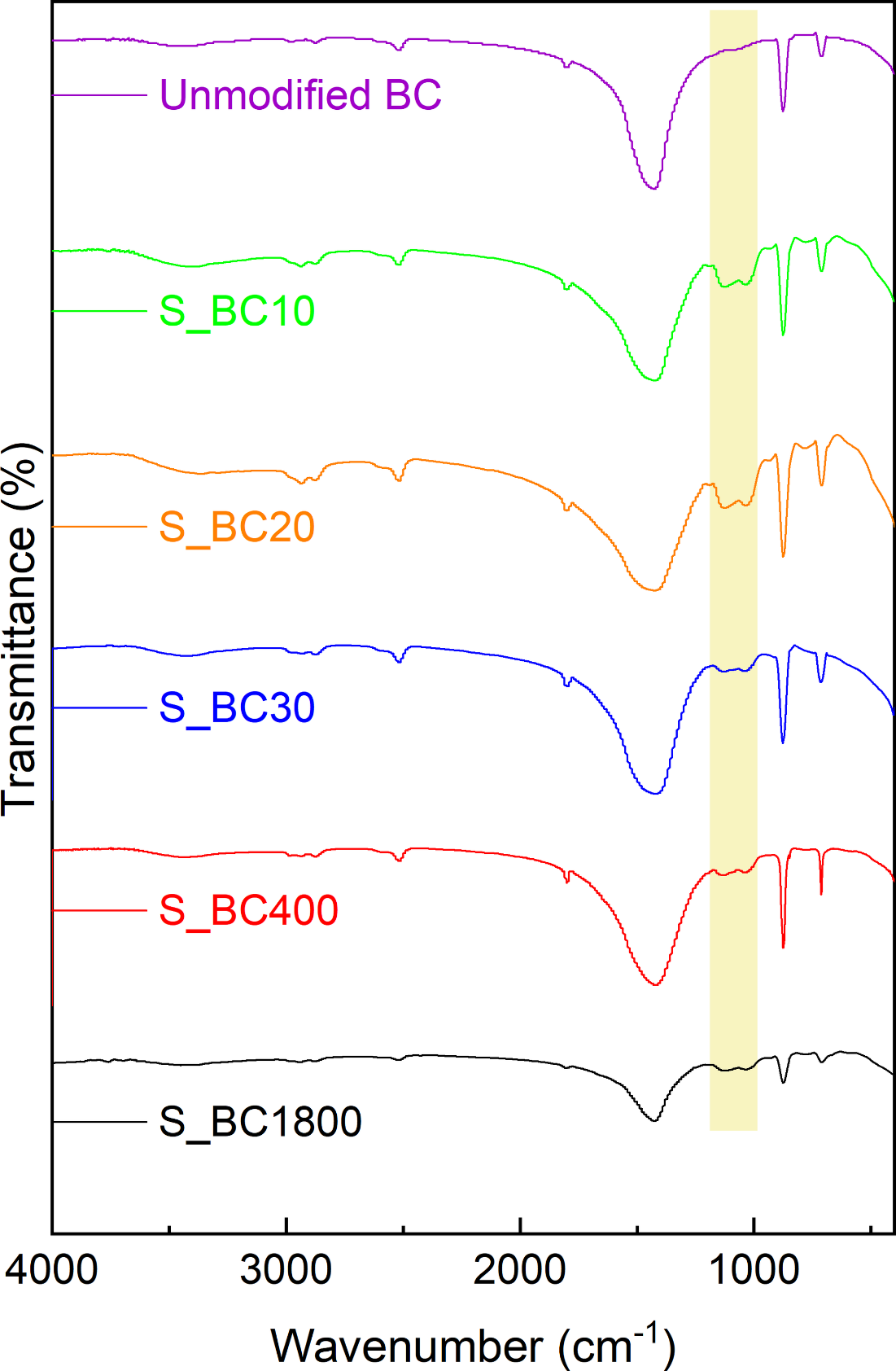


**Figure S4** Typical FTIR spectra of untreated BC and modified BC.


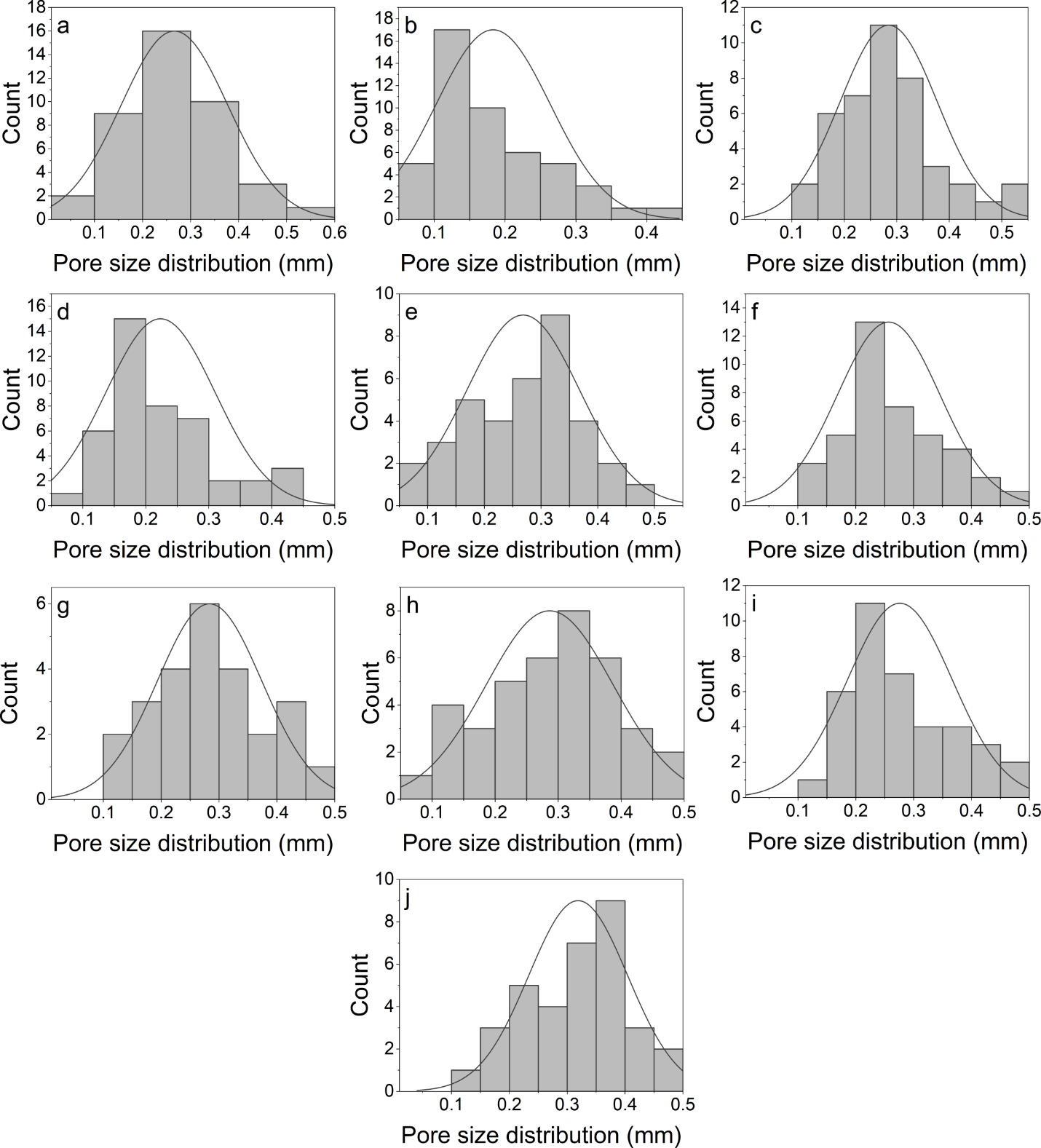


**Figure S5** Pore size distribution of (a) unfilled FPU, (b) FPU/BC0.1, (c) FPU/BC0.3, (d) FPU/BC0.5, (e) FPU/BC0.7, (f) FPU/S_BC10, (g) FPU/S_BC20, (h) FPU/S_BC30, (i) FPU/S_BC400, and (j) FPU/S_BC1800.


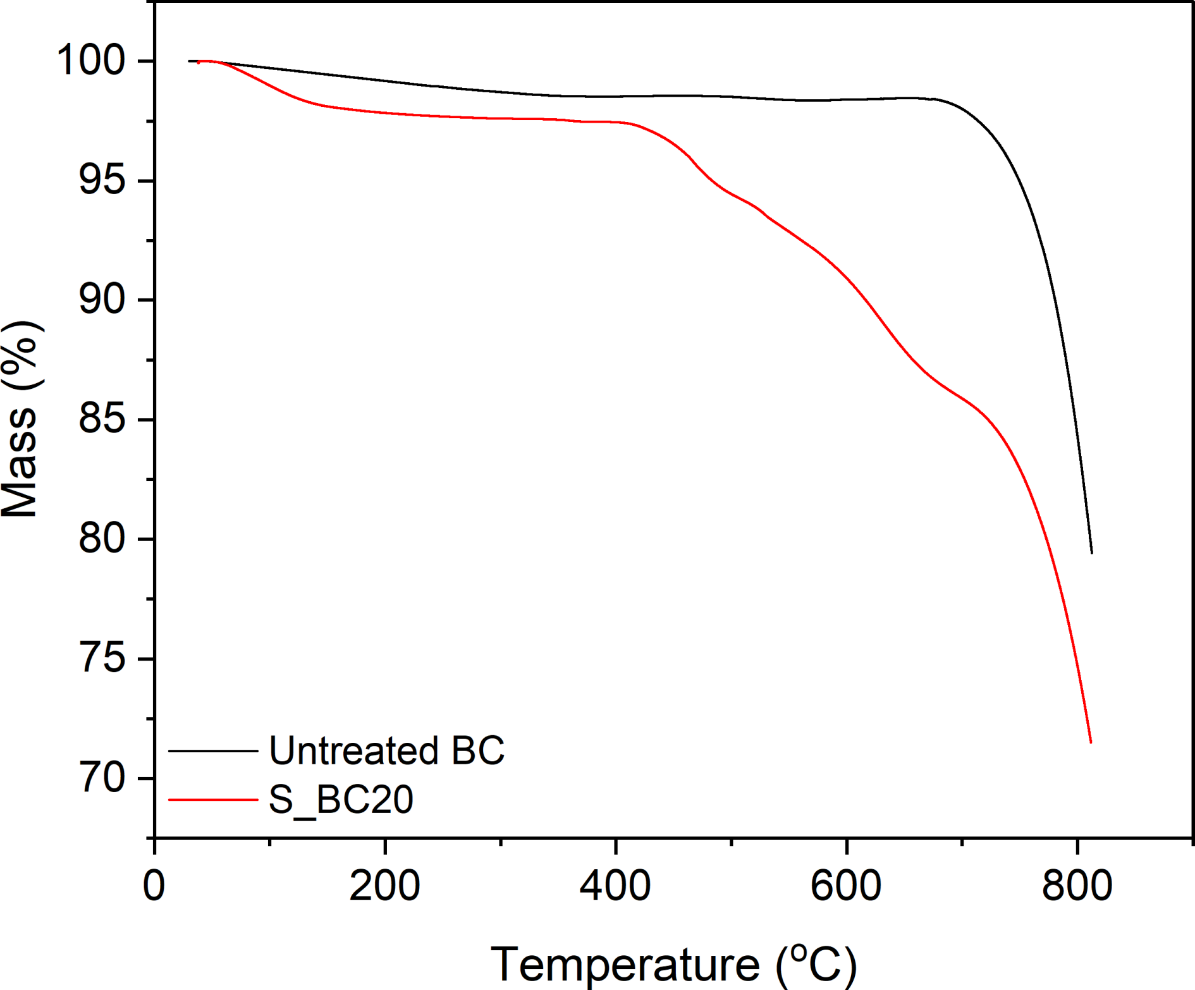


**Figure S6** TGA thermograms of untreated BC and S_BC20.
